# Dual RNA-seq study of the dynamics of coding and non-coding RNA expression during *Clostridioides difficile* infection in a mouse model

**DOI:** 10.1128/msystems.00863-24

**Published:** 2024-11-27

**Authors:** Victor Kreis, Claire Toffano-Nioche, Cécile Denève-Larrazet, Jean-Christophe Marvaud, Julian R. Garneau, Florent Dumont, Erwin L. van Dijk, Yan Jaszczyszyn, Anaïs Boutserin, Francesca D'Angelo, Daniel Gautheret, Imad Kansau, Claire Janoir, Olga Soutourina

**Affiliations:** 1 Université Paris-Saclay, CEA, CNRS, Institute for Integrative Biology of the Cell (I2BC), Gif-sur-Yvette, France; 2 Université Paris-Saclay, INRAE, AgroParisTech, Micalis Institute, Orsay, France; 3 Biomics Platform, C2RT, Institut Pasteur, Paris, France; 4 UMS IPSIT, Université Paris-Saclay, Orsay, France; 5 Institut Universitaire de France (IUF) 89211 , Paris, France; Vanderbilt University Medical Center, Nashville, Tennessee, USA

**Keywords:** dual RNA-seq, *Clostridioides difficile*, host-pathogen interactions, non-coding RNA, gut microbiota

## Abstract

*Clostridioides difficile* is the leading cause of healthcare-associated diarrhea in industrialized countries. Many questions remain to be answered about the mechanisms governing its interaction with the host during infection. Non-coding RNAs (ncRNAs) contribute to shape virulence in many pathogens and modulate host responses; however, their role in *C. difficile* infection (CDI) has not been explored. To better understand the dynamics of ncRNA expression contributing to *C. difficile* infectious cycle and host response, we used a dual RNA-seq approach in a conventional murine model. From the pathogen side, this transcriptomic analysis revealed the upregulation of virulence factors, metabolism, and sporulation genes, as well as the identification of 61 ncRNAs differentially expressed during infection that correlated with the analysis of available raw RNA-seq data sets from two independent studies. From these data, we identified 118 potential new transcripts in *C. difficile*, including 106 new ncRNA genes. From the host side, we observed the induction of several pro-inflammatory pathways, and among the 185 differentially expressed ncRNAs, the overexpression of microRNAs (miRNAs) previously associated to inflammatory responses or unknown long ncRNAs and miRNAs. A particular host gene expression profile could be associated to the symptomatic infection. In accordance, the metatranscriptomic analysis revealed specific microbiota changes accompanying CDI and specific species associated with symptomatic infection in mice. This first adaptation of *in vivo* dual RNA-seq to *C. difficile* contributes to unravelling the regulatory networks involved in *C. difficile* infectious cycle and host response and provides valuable resources for further studies of RNA-based mechanisms during CDI.

**IMPORTANCE:**

*Clostridioides difficile* is a major cause of nosocomial infections associated with antibiotic therapy classified as an urgent antibiotic resistance threat. This pathogen interacts with host and gut microbial communities during infection, but the mechanisms of these interactions remain largely to be uncovered. Noncoding RNAs contribute to bacterial virulence and host responses, but their expression has not been explored during *C. difficile* infection. We took advantage of the conventional mouse model of *C. difficile* infection to look simultaneously to the dynamics of gene expression in pathogen, its host, and gut microbiota composition, providing valuable resources for future studies. We identified a number of ncRNAs that could mediate the adaptation of *C. difficile* inside the host and the crosstalk with the host immune response. Promising inflammation markers and potential therapeutic targets emerged from this work open new directions for RNA-based and microbiota-modulatory strategies to improve the efficiency of *C. difficile* infection treatments.

## INTRODUCTION

*Clostridioides difficile* is an anaerobic spore-forming bacterium and the major cause of nosocomial infections associated with antibiotic therapy (1). The major risk factors to contract *C. difficile* infections (CDIs) are advanced age, the use of broad-spectrum antibiotics, and immune system deficiencies. The disruption of the colonic microbiota by antimicrobial treatments precipitates colonization of the intestinal tract by *C. difficile* and ultimately leads to infection. Increasing severe forms and high recurrence rates favored by persistent dysbiosis motivate the studies of *C. difficile* pathogenesis to develop synergistic and alternative treatments of CDI. Several *C. difficile* virulence factors have been identified, the toxins mainly responsible for epithelium lesions and clinical signs, as well as colonization factors like flagella and surface proteins. However, many aspects of *C. difficile* pathogenesis control remain poorly understood (2). Several bacterial factors, in particular, *C. difficile* toxins and flagella, have been described to activate the inflammatory response (3, 4), which aims to clear the pathogen but can also contribute to the severity of intestinal lesions through an uncontrolled inflammatory process. Better understanding the regulations of both host response and the bacterial virulence factor expression during the infection is essential to improve our understanding of this important human pathogen.

During infection, bacteria reprogram the expression of their genes in response to diverse environmental constraints. Intensive studies of bacterial transcriptomes have shown the presence of a large number of non-coding RNAs (ncRNAs) (5) participating in the regulation of adaptive and pathogenic processes (6, 7). Like in other pathogens, regulatory RNAs may shape virulence of *C. difficile*. Bioinformatics, RNA-seq, and genome-wide promoter mapping identified more than 200 ncRNAs of different functional classes in *C. difficile*, suggesting the diversity of RNA-based mechanisms for successful development of *C. difficile* inside the host (8–10).

Among them, several riboswitches responding to the signaling molecule c-di-GMP, coordinately control motility and biofilm formation, while multiple CRISPR (clustered regularly interspaced short palindromic repeats) RNAs are expressed to provide efficient defence against foreign genetic invaders for *C. difficile* survival in phage-rich gut communities (9, 11–13). Antisense RNAs act as antitoxins within type I toxin–antitoxin modules contributing to prophage stability (14–16) and *trans*-acting ncRNAs work in concert with the RNA chaperone protein Hfq to control the metabolic adaptations, biofilm formation, stress responses and sporulation (17–19).

From the host side, ncRNAs, including microRNAs (miRNAs) and long noncoding RNAs (lncRNAs), have been largely involved in the regulation of host inflammatory response and outcome of the infectious diseases (20). In general, ncRNAs and, in particular, the miRNAs, operate in a complex network. A global view of differentially expressed ncRNAs in the host during CDI is currently missing. Since the modulation of the host response dramatically impacts the clinical outcome (4), deciphering the role of the host ncRNAs will provide new perspectives to control severe forms and recurrences of CDI.

We used here a dual RNA-seq (21) for simultaneous monitoring of the host responses to infection and bacterial riboregulators involved in host–pathogen interactions, successfully adapted to various infection models (22–25). *In vivo* transcriptomics have been analyzed separately from pathogen or host side in several studies using microarrays or RNA-seq during CDI (26–29). The first *in vivo C. difficile* transcriptomic studies have been performed in a mono-associated mouse model of CDI (26, 29, 30), and the use of microarrays excluded the ncRNAs from this analysis. We explore here for the first time the dual transcriptome in a conventional *in vivo* model of CDI that better mimics the human infection to identify novel ncRNAs shaping *C. difficile* virulence and host response. As expected, several *C. difficile* virulence markers and host inflammatory response genes were induced during infection. Our dual RNA-seq analysis identified 61 ncRNAs among differentially expressed genes in *C. difficile in vivo* that could mediate the adaptation of *C. difficile* inside the host. From the host side, 185 ncRNAs were differentially expressed during infection, including numerous lncRNAs and miRNAs, enriching the regulatory network governing host response to pathogen infection. A particular gene expression profile from the host was associated with symptomatic CDI, paving the way for a better understanding of the process leading from colonization to symptomatic infection.

## MATERIALS AND METHODS

### Bacterial strains, growth conditions and preparation of spores

This work was performed with the *C. difficile* strain 630Δ*erm* (31), derived from the clinical 630 strain isolated from a patient suffering pseudomembranous colitis, widely used for ncRNA studies and mouse model experiments (27, 30, 32). This strain belongs to the PCR ribotype 012 that is considered by hospital-based survey as the eighth most common in Europe and also found among clinical strains in USA (33). For the *in vitro* culture, vegetative *C. difficile* cells were grown in brain heart infusion (BHI) at 37°C in an anaerobic chamber (5% H_2_, 5% CO_2_, 90% N_2_, Jacomex, France) during 8 h to reach late exponential growth phase (OD_600_ around 1.5). For the mouse challenge, *C. difficile* spores were prepared as previously described (34). Vegetative cells were eliminated by heating at 70°C during 25 min, and spores were numerated on BHI solid medium supplemented with taurocholate (0.1%) incubated 48 h at 37°C under anaerobic conditions.

### Animal model and treatment

All animal assays were conducted in accordance with the institutional guidelines that follow the European Union guidelines for the handling of laboratory animals. All procedures of the protocol were approved by the Committee on the Ethics of Animal Experiments C2EA-26 (n° APAFIS#4617–2016032118119771v1) of the Paris-Saclay University and the French Ministry of Research. All efforts were made to minimize animal suffering.

**Fig 1:**
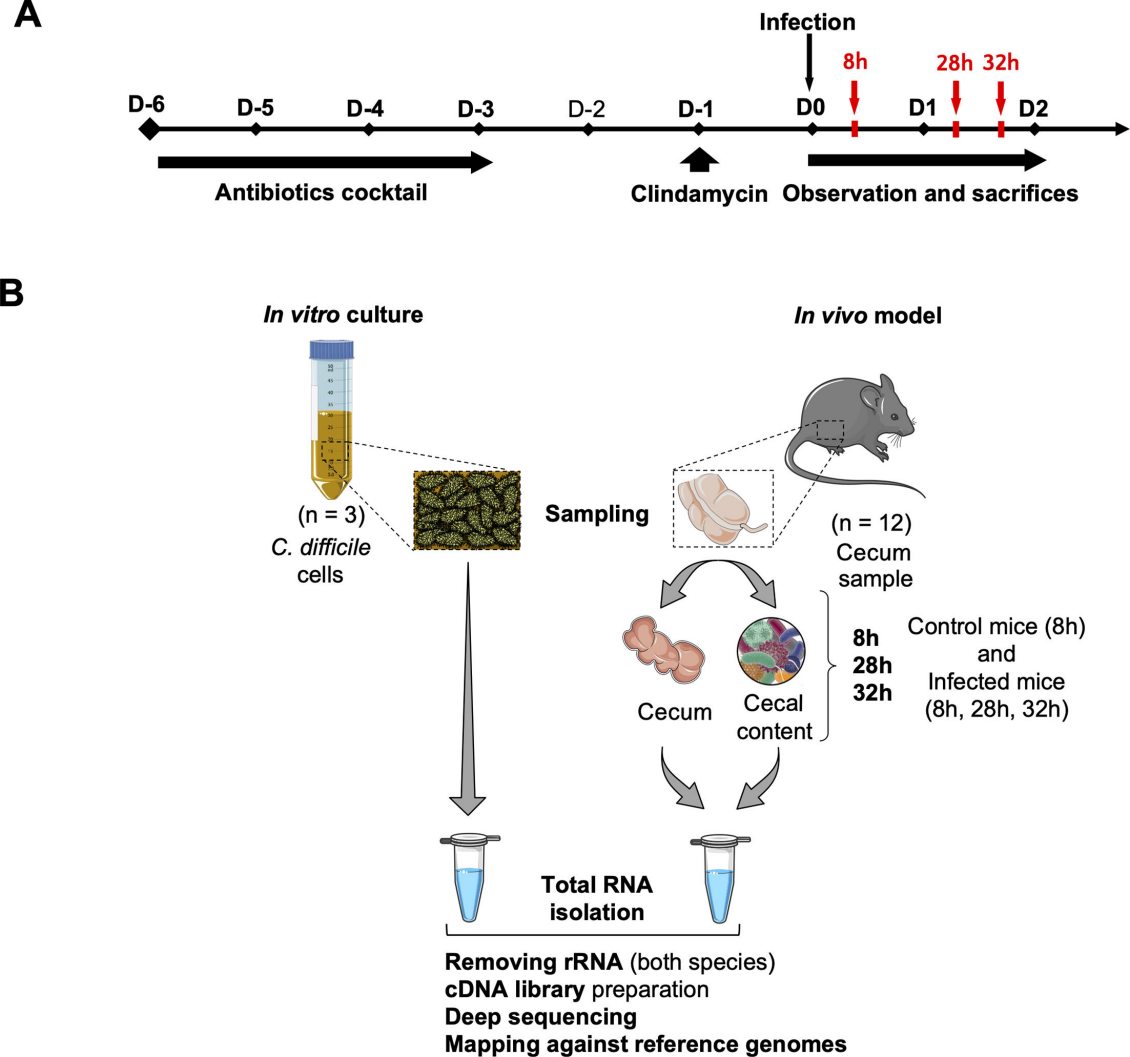
Description of dual RNA-seq experiment workflow. (**A**) The mouse model for *C. difficile* infection used for dual RNA-seq experiment has been described previously by Chen et al. (35). All the mice, including the control mice, received the same antibiotic treatment prior to the infection. Nine mice were infected with a suspension of 10^5^ spores of the *C. difficile* 630Δ*erm* strain, and three control mice received sterile water. Three infected mice were euthanized at each time point 8, 28, and 32 h post-infection for RNA extraction. The three uninfected control mice were euthanized 8 h after the administration of water. (**B**) All the mice (infected and uninfected) were treated according to the same protocol. For each mouse, the RNAs from the cecum and its content were extracted and purified separately before being assembled in one tube and then sequenced together. RNAs from the *in vitro* culture cells were purified with the same protocol (TRIzol) and sequenced with the same method (Illumina).

Twelve 6- to 7-week-old conventional C57BL/6 female mice were acquired from Charles River Laboratories (France) and were housed at the animal facility of the Faculty of Pharmacy, Paris-Saclay University (agreement number 92-019-01), with *ad libitum* access to irradiated food and autoclaved water. To induce an intestinal dysbiosis allowing their infection by *C. difficile,* mice received an antibiotic pre-treatment (35) (Fig. 1A) (Supplementary methods). Mice were infected by oral gavage with 10^5^ spores each, whereas mice from the control group received water and were co-housed by treatment groups of three animals (Supplementary methods). Three mice from the infected groups were euthanized 8, 28, and 32 h post-infection, and the three uninfected mice were euthanized at 8 h. Following sacrifice, entire ceca were collected, and cecal content was sampled to determine the burden of *C. difficile* (Fig. 1B). The rest of the ceca and their contents were placed in RNA*protect* solution (Qiagen) for further RNA extraction (Supplementary methods).

### RNA extraction, library preparation, and RNA sequencing

For *in vivo* RNA isolation, eukaryotic and prokaryotic cells were lysed separately in Fast-prep apparatus (lysing matrix D and B, respectively, two cycles of 30 s at speed 6.5). Total RNA isolation was then performed using Trizol (Sigma) as described previously (36) (Fig. 1B). The quality of eukaryotic and prokaryotic total RNAs was tested with Agilent RNA 6000 Pico kit and quantified before sequencing. For library preparation, eukaryotic and prokaryotic RNAs were mixed in a 2:1 proportion to ensure sufficient genome coverage and maximize the prokaryotic RNA sequencing. Ribosomal RNA depletion and library preparation were done using an Illumina TruSeq Stranded Total RNA kit with 1:1 mixture of human/mouse/rat and bacterial rRNA removal solutions for rRNA depletion. The resulting libraries were multiplexed and sequenced on an Illumina NextSeq 500 system as a paired end (PE) 50–35 nucleotide run using a NextSeq 500/550 High Output 75 cycles v2 reagent kit (Illumina).

### Reads alignment and differential expression analysis

The sequencing data were processed as described in Supplementary methods. Differential expression analysis was performed using the DESeq2 (37) based script SARTools (38), and genes were considered differentially expressed with at least 1 log_2_ fold change and an adjusted *P* < 0.05. All analysis for mouse transcriptome data were performed using R (39) and RStudio (40). Genes with count mean lower than 10 were discarded for downstream analyses (39,719 to 21,085 genes). Trimmed mean of M values (TMM) normalization was done using edgeR (41), and data were linearized with voom function from limma with quantile normalization (42, 43). Then, we applied a one-way analysis of variance for TREATMENT factor for each gene and made pairwise Tukey’s *post hoc* tests between groups. Significant gene with *P* < 0.05 and fold-change >2 (or <−2) in at least one comparison were selected for downstream analysis (2,297 genes). For functional enrichment analysis, we used MSigDB v7.5 (43) and applied Fisher exact test with false discovery rate (FDR) correction for multiple testing to find significant overlaps.

### Functional classification with MA2HTML

Gene-set enrichment was assessed using functional gene classification from the Ma2HTML database (44) (https://mmonot.eu/MA2HTML/, extraction number 1652263129), and the log_2_ fold change values between *in vivo* (MI) versus *in vitro* (IV) conditions of each of the 2,855 genes with an associated functional class. For the 20 classes, gene-set enrichment was measured with the blitzGSEA software (45).

### Taxonomic classification of shotgun sequencing reads

Relative bacterial cell abundances were evaluated using mOTUs2 (46). Briefly, mOTUs2 performs taxonomic classification of shotgun metagenomics and metatranscriptomics sequencing reads using a single-copy, non-16s rRNA marker gene approach. The 10 marker genes used are highly conserved and can serve as good proxies to assess the relative abundances of active cells in the community. Prior to classification, mouse reads were removed from the data set by two consecutive alignments to the mouse genome (RefSeqs accession GCF_000001635.27) using STAR (47) and Bowtie2 (48). mOTUs2 was run on each sample read data set using the following parameters: $ motus profile -s R1_001.fastq -t 10k taxonomic_level -B -q -o output_file_ABOND.motus. Intergroup differences at the species level were assessed using the linear discriminant-analysis effect size (LEfSe) method (49). LEfSe uses Kruskal–Wallis test (two-tailed nonparametric) and Wilcoxon rank-sum test to determine the level of significance of differences in features (bacterial taxa) between two conditions. Clades/taxa with an LDA score ≥2 and alpha value for the Wilcoxon test ≤0.05 were considered significantly different between compared groups.

### Analysis of available *in vivo* transcriptomics data for differential ncRNA expression

For comparative analysis with available *in vivo* raw transcriptomics data from previously reported data sets, we chose the conditions that most closely approximated the comparison we realized during the present study: *in vivo* conditions in infected mice vs *in vitro* conditions. We used the *in vivo* WT_RNA 3-days post*-C. difficile* infection (European Nucleotide Archive, ENA identifier: PRJNA666929) vs *in vitro* base media (ENA identifier: PRJNA667108) data reported by Pruss et al. (32) and day 2 post*-C. difficile* infection vs *in vitro* overnight culture in TY medium (ENA identifier: PRJNA612095) in Fletcher et al. (27). All RNA-seq replicates of each condition were downloaded from ENA (see Table S5) and processed separately for the two experiments with a snakemake script (50) on the high-throughput computing resources of the French Institute of Bioinformatics (IFB) (Supplementary methods). Final tests and Fig. S9 were produced from differential gene analysis tables using a dedicated R script (with the Eulerr package [51]). The convergence of differential expression experiments was assessed using *χ^2^* tests on differential gene lists. Heatmaps of differential genes in all experiments were drawn (Complex Heatmap R package [52]) in log_2_ fold change unit, with values set to 0 in case of the absence of differential expression. All codes are available on github [https://github.com/i2bc/Dual_Seq_Cdiff_Mouse].

### NcRNA detection from RNA-seq data using DETR’PROK

The prediction of new RNAs was carried out using shell version 2.1.3 of the DETR'PROK pipeline (53) for two conditions of the three experiments with 630 *C*. *difficile: in vitro* and infected mice samples kIV, kMI from the present Kreis et al*.* study, *in vitro* base media (pBase), and *in vivo* WT_RNA 3-day post-infection (pWT) samples from Pruss et al. (32), and *in vitro* TY (fwtTY) and 2-day post-infection (fwtd2) samples from Fletcher et al. (27) (Supplementary methods and Table S6). DETR'PROK predictions for ncRNA for the six conditions were combined (clusterize.py, S-MART tools [54]), and candidates overlapping in sense or antisense (CompareOverlapping.py, S-MART tools) with previously annotated rRNAs or tRNAs were removed, yielding 118 potential new transcript candidates.

## RESULTS AND DISCUSSION

### Animal model and protocol optimization

Although several *in vivo* transcriptomic studies have already been carried out on *C. difficile* and mice separately (26–29, 32, 55), none have so far looked by RNA-seq in animal model at the expression dynamics of RNAs including ncRNAs simultaneously in the pathogen and the host. We have chosen the conventional mouse model that mimics the conditions of human infection associated with antibiotic pre-treatment in animals (35). To determine optimum sampling time points after infection to both recover sufficient cecal content for bacterial RNA extraction and to observe the onset of clinical signs for host response, we first set up a clinical follow-up assay on six conventional mice that were infected with 630Δ*erm* spores. In this validated model, the clinical signs of CDI appear between 24 and 36 h post-infection. After the oral challenge, mice were monitored during 40 h to check the occurrence and evolution of the symptoms induced by CDI, the colonization rate, and weight of the infected mice. The animals were observed regularly for signs of disease, including the consistency of their stools and their behavior, and weighed once a day to account for normal fluctuations, and a clinical sickness score (CSS) has been established (Supplementary methods and Fig. S1). The first symptoms appeared at 32 h with soft stools in all mice, reduced activity, and hunched posture in half of the mice. The peak of symptoms was observed at 40 h post-infection, with a CSS score of 6–7; all mice had a marked weight loss, and ceca showed a hemorrhagic appearance indicating inflammation. The *C. difficile* colonic colonization plateau was reached at 8 h post-infection with approximately 10^8^ vegetative forms/g of feces and starts to decrease with the appearance of diarrhea leading to the loss of almost all the cecal content at 40 h post-infection. We then selected three time points in the infection kinetics for RNA extraction: an early 8 h post-infection point associated to the colonization plateau, and two late 28 and 32 h post-infection points where the first symptoms appear associated with an immune response engaged, but when there is still sufficient quantity of cecal content for sampling before excessive diarrhea.

For dual RNA-seq, three groups of mice (8, 28, and 32 h post-challenge) (Fig. 1A) were infected with *C. difficile* spores, and the control mice received saline water solution. At each sampling time point, mice were euthanized, and their ceca were sampled. Quantification of *C. difficile* cecal burden confirmed the host colonization by an average of 10^8^ CFU/g of feces of *C. difficile* vegetative cells at the three time points tested (Table S1). We observed diarrhea and a lack of activity for three infected mice in two different groups: one mouse after 28 h of infection and two mice after 32 h. These mice also had a smaller, less filled, and inflamed cecum compared with the other mice. The mouse ceca and contents were lysed separately followed by RNA extraction, yielding two RNA samples for each mouse, predominantly eukaryotic or predominantly prokaryotic RNA (averaging 1,200 ng/µL and 120 ng/µL, respectively). Samples were mixed for each mouse prior to sequencing in controlled proportions to maximize the amount of prokaryotic RNA sequenced.

### Dual RNA-seq analysis of *C. difficile* infected mouse cecum

The distribution of reads mapped on reference genomes for each group are illustrated in Fig. S2. The total RNAs for host cecal tissue and microbial cecal content containing pathogen were isolated from infected mice at 8, 28, and 32 h post-infection and then analyzed by Illumina deep sequencing. The number (*n* = 3) of *C. difficile* and mouse PE reads from 8, 28, and 32 h post-infection mapped on the reference genomes (GCF_000009205.2 and GCF_000001635.27) are indicated in Fig. S2A and B, respectively. The remaining PE reads were kept for gut microbiota composition analysis (Fig. S2C). Finally, total RNAs were also extracted from three independent 8 h *in vitro* cultures of *C. difficile* 630Δ*erm* strain and sequenced (Fig. S2A). Given the large gap of reads number between the *C. difficile in vitro* and *in vivo* samples, a representative subsampling of 0.5% of *in vitro* reads has been performed to allow their normalization with the *in vivo* samples for further differential gene expression analysis. Due to the low number of reads in the samples extracted at 8 h post-infection, only the data obtained at 28 and 32 h were retained for differential *C. difficile* gene expression analysis. We decided to combine these samples together for the comparison with the *in vitro* condition since no significant difference between these two data sets was observed. The principal component analysis (PCA) also revealed similarity between these samples, which are 78% separated from the *in vitro* samples by the PC1 axis (Fig. S3).

### Microbial community abundance profiling from dual RNA-seq data using mOTUs2

**Fig 2:**
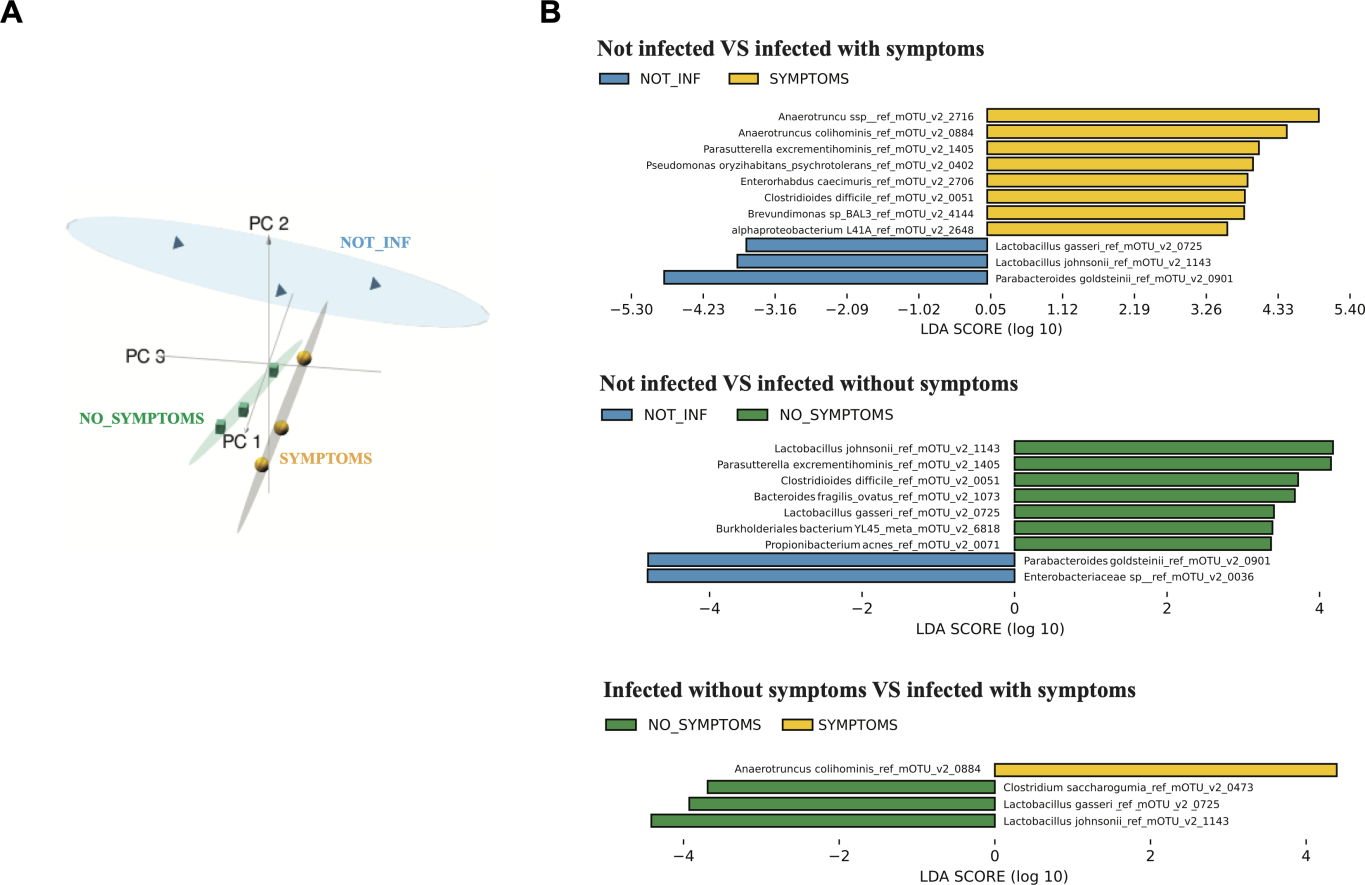
Principal component analysis of the bacterial species identified in the mouse gut (**A**) and analysis of biomarkers between conditions using LEfSe (**B**). (**A**) Each symbol on the three-dimensional PCA score plot represents a sequenced mouse gut sample. Symbols are colored according to experimental conditions (blue pyramids = NOT_INF: mice not infected with *C. difficile*, yellow spheres = SYMPTOMS: mice infected with *C. difficile* showing visible symptoms, green cubes = NO_SYMPTOMS: mice infected with *C. difficile* showing no visible symptoms). The three main principal components and the corresponding variance proportion are shown. (**B**) Histogram of the LDA scores computed for features differentially abundant between the different conditions being compared. LEfSe score indicates the consistent difference in relative abundance between the features (taxonomic groups) in the microbial communities. The histogram shows which clades/taxons among all those detected are statistically significant between the conditions compared. Only clades/taxa with an LDA score ≥2 and reaching significance are shown (alpha value for the Wilcoxon test ≤0.05).

Microbiota composition constitutes a key parameter affecting the development of *C. difficile* inside the gut and individual outcome of infection. Here, we took advantage of conventional mouse model to look at the microbiota composition during CDI using dual RNA-seq data. We applied mOTUs2 program well-adapted for taxonomic profiling of microbial community on housekeeping marker genes from transcriptomic data (46). As for differential analysis of *C. difficile* gene expression, 8 h post-infection samples have been excluded from these microbiota analyses. The PCA of bacterial species in mouse gut revealed a cluster of uninfected mice samples clearly separated from infected samples (Fig. 2A). In the group of infected mice, two clusters could be distinguished corresponding to the groups of *C. difficile*-infected mice presenting or not visible symptoms, clearly seen in three-dimensional PCA graph (Fig. 2A). On loading PCA plot (Fig. S4A), *C. difficile* appears as a discriminant bacterium in infected conditions, contributing as expected to the sample separation. Indeed *C. difficile* modulates the composition of microbiota, either directly *via* production of *p*-cresol (56) or in an indirect way by inducing indole production by *E. coli* (57).

The relative community composition at different taxonomic levels is shown in Fig. S5. This metatranscriptomic analysis revealed profound alterations in the structure of mouse gut microbiota associated with *C. difficile* colonization and inflammatory symptoms in infected mice, as previously described in other mouse models of CDI (58, 59). In humans, some common features of dysbiosis have been found in microbiota studies of patients suffering from CDI (58–61). To search for additional discriminating taxa at species mOTUs level as biomarkers associated with CDI symptoms in our study, we used linear discriminant analysis (LDA) effect size (LEfSe) method (49). The histograms presented in Fig. 2B show the clades identified as significantly different between compared conditions that explain the greatest differences between analyzed microbial communities. All pairwise comparisons identified as expected *C. difficile* as overabundant species in the infected mice community (Fig. 2B). *Lactobacillus* species including *Lactobacillus gasseri* and *Lactobacillus johnsonii* were enriched both in uninfected and symptom-free mice as compared to symptomatic mice and identified as discriminating between non-symptomatic and uninfected samples, suggesting a potential positive role of *L. gasseri* and *L. johnsonii* in inhibition of CDI symptom development (Fig. 2B; Fig. S4B). *Lactobacillus reuteri* was greatly depleted in two symptomatic mice samples 32 h post-infection (Fig. S4B). Several strains of *L. reuteri* have been previously shown to inhibit *C. difficile* growth *in vitro* (59, 62) but also *in vivo* (63). In contrast, *L. johnsonii* does not seem to have an effect on the growth of *C. difficile* (63), but its protective effect highlighted in our study could be related to its anti-inflammatory properties (64). Our analysis also revealed a significant association of *Clostridium saccharogumia* with non-symptomatic infected mice as compared with symptomatic mice, and a significant association of *Alistipes indistinctus* with uninfected mice as compared with infected mice (Fig. 2B; Fig. S4B and S5A). The *Alistipes* genus was already associated with a protective effect against CDI in a mouse model (65), being an important post-fecal microbiota transplantation (FMT) genus in humans (66). Relevant to previous observations (27, 67–69), in our study, several *Bacteroidales* species (*Bacteroides dorei/vulgaris* and *B. fragilis*) have been enriched in non-symptomatic infected mice (Fig. S4B). In contrast, a group of *Anaerotruncus, Enterorhabdus,* and *Brevundimonas* species was identified as positively associated with *C. difficile* in infected samples, in particular, in symptomatic mice and as depleted in uninfected samples (Fig. 2B; Fig. S4B). To our knowledge, these genera have not been previously associated, positively or negatively, to *C. difficile*, except for *Anaerotruncus colihominis*. Surprisingly, this Gram-positive rod-shaped bacterium is known to have anti-inflammatory effects, probably by butyric acid production and was long-term increased after FMT in one patient suffering from CDI (70). Thus, this species deserved more studies to understand the nature of its potential interaction with *C. difficile*.

This microbiota composition profiling allowed clustering the samples into three groups (uninfected mice, symptomatic, and non-symptomatic infected mice) to consider during the course of this study exploring the correlation between microbiota structure, *C. difficile* gene expression and mice transcriptomic data.

### Comparative *C. difficile* transcriptomic analysis between *in vitro* culture and infectious conditions

**Fig 3:**
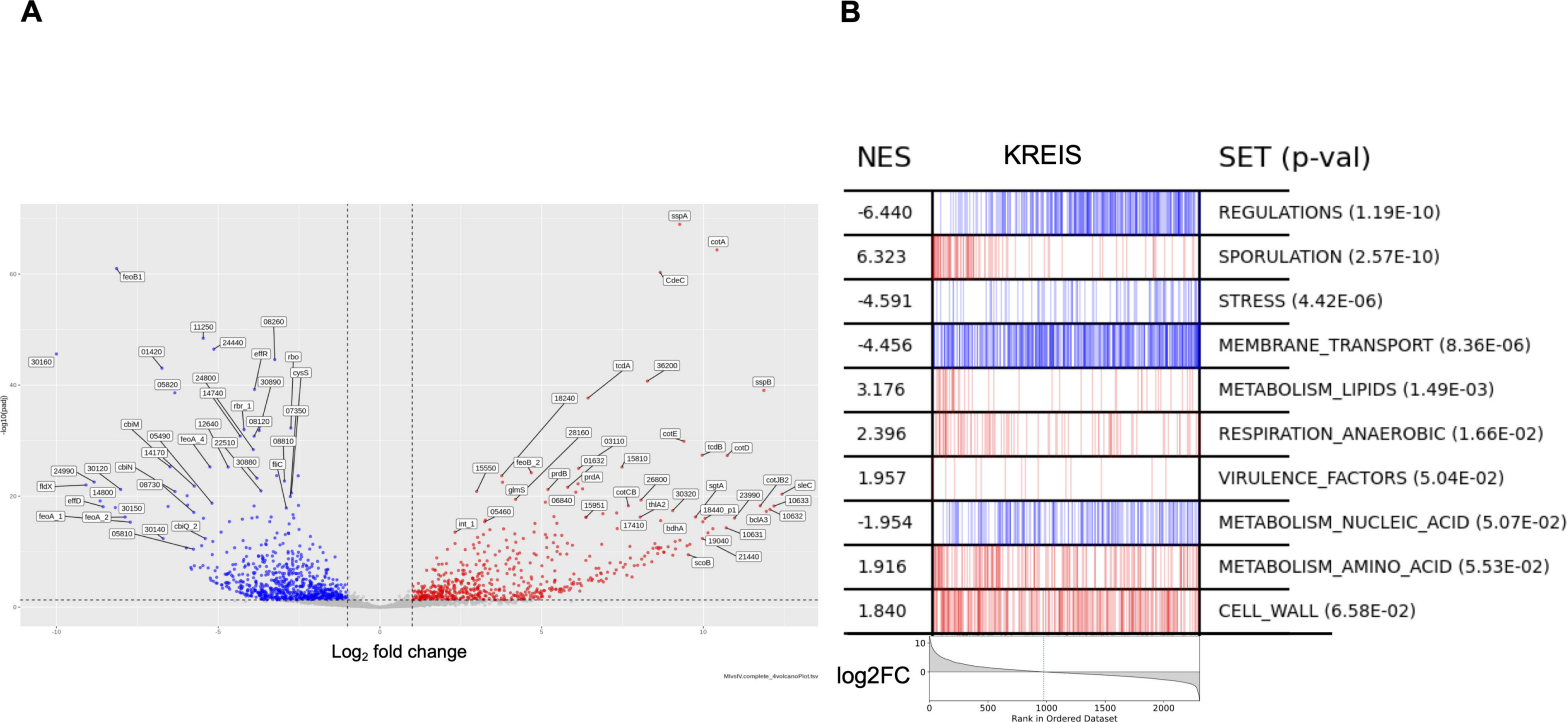
Genes of *C. difficile* differentially expressed between groups of mice infected at 28 and 32 h post-infection (MI) and *in vitro* cultures (IV). (**A**) Volcano plot representing the logarithm of the *P*-value adjusted according to the logarithmic ratio (log_2_ fold change; FC) of the genes differentially expressed between the two conditions. The colored dots correspond to the genes significantly differentially expressed (the genes induced or repressed under the MI conditions in red and blue, respectively). (**B**) Enrichment analysis of Ma2HTML classes with *C. difficile* expression profiles in infected mice versus *in vitro* growth conditions. The enrichment score reflects the concentration on one side of the genes belonging to the class (left side, red for upregulated differentially expressed genes; right side, blue for downregulated differentially expressed genes) as the genes are ordered according to their decreasing log_2_FC (gray curve at the bottom). NES: normalized enrichment score; SET: class name; *p*-val: adjusted *P*-value.

For *C. difficile*, our transcriptomic analysis revealed a total of 1,309 genes (559 upregulated and 750 downregulated) (Table S2) exhibiting differential expression between *in vitro* and a 28–32h post-infection condition (Fig. 3A), including 61 ncRNA genes (Table 1). All differentially expressed genes were then assigned to functional categories with the MA2HTML database classification (Fig. 3B). Gene-set enrichment analysis was performed to compare *C. difficile* gene expression profiles in infected mice and *in vitro* growth conditions. Eleven classes out of 20 have an adjusted *P*-value < 0.05 associated with an FDR <25% (Table S3). The two classes showing the best normalized enrichment score are associated with sporulation as upregulated gene set and regulations as commonly downregulated class (Fig. 3B; Fig. S6).

**TABLE 1:** List of *C. difficile* ncRNAs differentially expressed during infection compared with *in vitro* culture

Gene	Log2 fold change	*P*adj	Product name
CD630_SQ1296	8.185	6.45E-10	ncRNA IGR
CD630_SQ995	7.037	4.98E-07	ncRNA IGR
CD630_SQ1076	6.37	6.52E-17	Antisense 3'UTR
CD630_s0040	5.405	1.82E-05	5'UTR *glmS*
CD630_s0460	5.316	0.00028877	Lysine riboswitch
CD630_n00930	5.16	2.55E-05	ncRNA IGR
CD630_RNA_11	4.808	0.01105347	SCARNA14
CD630_s0631	4.767	2.90E-14	T-box *CD630_32560*
CD630_s0280	3.676	0.00066216	T-box leader
CD630_n00640	3.584	3.42E-08	ncRNA IGR
CD630_SQ2503	3.536	0.01479877	ncRNA IGR
CD630_SQ2429	3.075	0.01139522	ncRNA IGR
CD630_SQ1038	2.956	0.02238772	ncRNA IGR
CD630_Cdi1_11	2.952	0.00567505	GEMM RNA motif
CD630_s0281	2.89	0.00984163	5'UTR *hisZ*
CD630_n00680	2.871	5.75E-06	ncRNA IGR
CD630_Cdi1_6	2.855	6.85E-06	GEMM RNA motif RCd5
CD630_s0210	2.755	0.02952147	T-box (Leu)
CD630_s0590	2.571	0.00182813	Lysine riboswitch
CD630_s0540	2.527	3.21E-05	T-box (Trp)
CD630_SQ808	2.524	0.0006825	Antisense 3'UTR
CD630_n00430	2.466	0.00163311	Antisense CDS
CD630_s0660	2.458	0.00262189	T-box (Met)
CD630_s0610	2.318	0.00032484	Lysine riboswitch
CD630_s0642	2.222	0.01345762	5'UTR putative zinc finger protein gene
CD630_n00620	2.135	1.43E-05	ncRNA IGR
CD630_s0070	2.058	1.41E-08	Purine riboswitch
CD630_s0270	2.053	0.00117689	T-box leader
CD630_SQ1005	2.053	0.00117689	ncRNA IGR
CD630_n00410	2.039	6.63E-07	ncRNA RCd3
CD630_n00350	1.978	1.46E-06	ncRNA IGR
CD630_s0550	1.911	0.00677355	T-box (Ile)
CD630_s0010	1.871	0.00183516	T-box (Ser)
CD630_n00560	1.652	0.02091479	ncRNA IGR
CD630_s0190	1.648	9.57E-05	T-box
CD630_SQ408	1.629	0.00017077	ncRNA IGR
CD630_Cdi1_8	1.537	0.0122029	GEMM RNA motif
CD630_s0480	1.253	0.04976858	T-box (Thr)
CD630_n00900	1.194	0.00107517	Antisense CDS
CD630_s0570	0.655	0.03940458	RNase *P*
CD630_n00990	−1.075	0.02099668	ncRNA IGR
CD630_n00380	−1.166	0.02571729	ncRNA IGR
CD630_n00290	−1.681	0.03236452	ncRNA IGR
CD630_n00450	−2.429	0.00184353	Antisense CDS
CD630_Cdi1_12	−2.695	2.84E-08	GEMM RNA motif
CD630_RNA_8	−2.904	0.0122029	PreQ1
CD630_n01080	−3.111	0.04001284	Antisense CDS
CD630_n00590	−3.483	0.04467695	ncRNA IGR
CD630_n00890	−3.592	0.03720248	Antisense CDS
CD630_n00950	−3.656	0.01282977	Antisense CDS
CD630_n00080	−3.679	0.02703932	Antisense CDS
CD630_n01100	−3.697	0.00204175	ncRNA IGR
CD630_s0220	−3.786	0.00221948	SAM riboswitch (S_box leader)
CD630_n00770	−3.821	6.82E-05	ncRNA IGR
CD630_n00170	−3.828	0.01565047	ncRNA RCd6
CD630_Cdi2_4	−3.891	9.79E-07	c-di-GMP-II
CD630_RNA_1	−4.191	0.00449118	PyrR
CD630_s0670	−4.278	0.00041904	5'UTR *luxS*
CD630_Cdi1_2	−4.294	0.000513	GEMM RNA motif
CD630_SQ367	−4.867	0.00037504	ncRNA IGR
CD630_n00830	−6.55	7.04E-19	Antisense CDS

**Fig 4:**
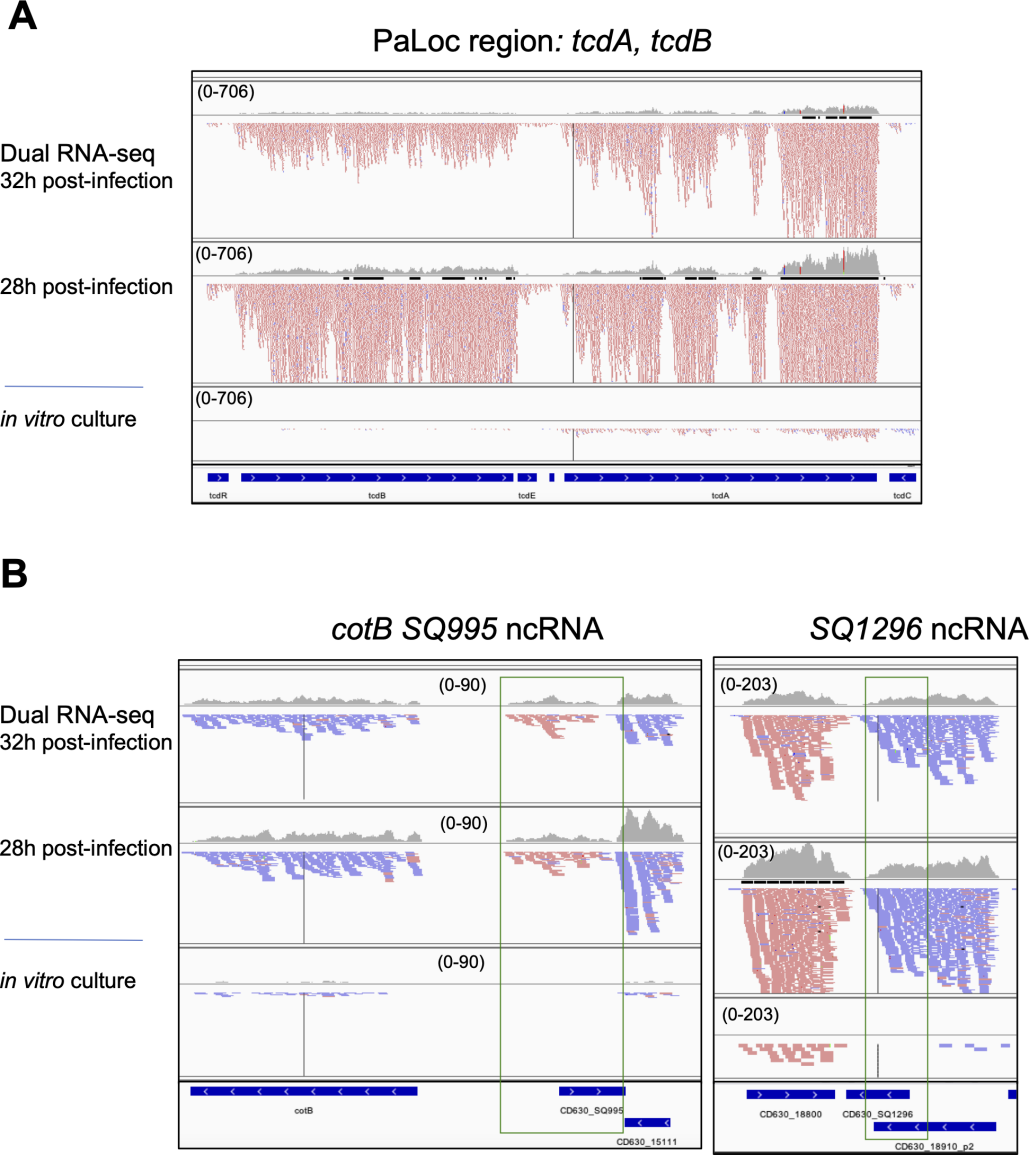
Visualization of dual RNA-seq data for *C. difficile* differentially expressed genes with IGV. Representative examples are presented in (**A**) for protein encoding genes including virulence factors TcdA and TcdB, in (**B**) for ncRNAs. The results for *in vivo* samples 28 and 32 h post-infection are compared with the data from *in vitro* sample. The genomic regions for ncRNAs are presented in a green box. Genes from MaGe annotation are shown at the bottom of each panel. In the IGV visualization profiles, “+” strand reads are shown in red, and “-“ strand reads are shown in blue. IGV visualization is presented with adjusted read threshold for each window to compare the data from different samples (scale is indicated as a read threshold range).

Among the genes induced during infection (Table S2) were numerous ribosomal genes reflecting high cellular activity *in vivo* with constant nutrient turnover. The induction of virulence factors, such as the toxins TcdA and TcdB (Fig. 4A) as well as the adhesin CwpV, promoting self-aggregation and phage resistance of *C. difficile* (71, 72) was in accordance to previous *in vivo* transcriptomics in mice (26, 29, 73) or in pigs (74). We validated the overexpression of *tcdA* gene in *C. difficile*-infected mice as compared with the control by independent qRT-PCR experiment (Fig. S7A). In accordance with different expressions of flagellar operons in a clinically relevant heat stress (75), some genes from the flagellar assembly F3 operon were induced, but several F1 flagellar operon genes were repressed *in vivo* compared with *in vitro* culture. The expression of most of the type IV pilus synthesis genes is decreased *in vivo* in accordance with inverse regulation of flagellum and *pilus* expression by antagonistic type I and type II c-di-GMP-dependent riboswitches (8, 9, 76, 77).

The adaptive metabolic capabilities of *C. difficile* are a fundamental part of the infectious process (30). One of the main sources of energy for *C. difficile* comes from the fermentation of carbohydrates and amino acids as an important asset to colonize its niche. Many genes dedicated to carbohydrate transport and metabolism are differentially expressed during infection, including the induction of 10 genes of phosphoenolpyruvate-dependent phosphotransferase system (PTS) for the acquisition and phosphorylation of sorbitol, fructose, mannitol, and galactitol, and the repression of about 20 genes for the transport of other sugars, such as mannose, lactose, but also glucose and glucosides. These sugars are probably absent in the cecum of mice, implying the preferential use of other carbon sources. For example, glucose is totally absorbed in the upper part of the intestine and the dysbiosis induced by antibiotics must considerably limit the degradation of complex fibers contained in the mice’s diet into monosaccharides.

The final stage of glycolysis leads to the production of pyruvate, which is then metabolized by several fermentative pathways for energy production or anabolic reactions. Some of these pathways, which lead to the production of butyrate, ethanol, or butanol, pass through a major intermediary product of bacterial carbon and energy metabolism, acetyl-CoA. Acetyl-CoA is the final degradation product of ethanolamine, an abundant compound *in vivo* that can be derived either from the degradation of cell membranes because of disease or directly from the diet. Remarkably, all genes of the *eut* operon involved in ethanolamine metabolism were strongly overexpressed in mice compared with the *in vitro* condition (Fig. S8A). Ethanolamine is a source of carbon and nitrogen for the bacteria. The expression of *eut* operon is repressed by glucose, and the observed overexpression is relevant with the repression of glucose transport systems.

*C. difficile* uses amino acids as an energy source through Stickland reactions for coupled fermentation of two amino acids acting as an electron donor and acceptor, respectively. Several amino acids can be used in the oxidative branch, the only acceptors are glycine and proline. We observed a strong overexpression of both selenoenzyme operons, the proline reductase *prd* (Fig. S8A) and the glycine reductase operon. Availability of proline in the host has been shown to modulate the bacterial capacity to infect the mouse (78), and proline and hydroxyproline are major components of collagen that can be released by its degradation to further sustain the growth of *C. difficile* (79).

The acquisition of iron from the environment is vital for most prokaryotes. We observed an overexpression of one of the three *feo* operons (*feo*2) (Fig. S8A) involved in the uptake of ferrous iron in many pathogenic bacteria. However, in *C. difficile*, this *feo* operon is neither under the regulation by the iron level nor the global regulator Fur (80, 81). In *Porphyromonas gingivalis,* an homologous Feo system is involved in manganese import, suggesting that this system could allow the uptake of Mn also in *C. difficile*, with a possible modulation for bacterial virulence since Mn is a cofactor of the toxins A and B. The other genes involved in iron acquisition, notably the ABC transporters capable of transporting ferric iron (*CD630_29970–29990*) are repressed *in vivo*. A recent study identified a particular iron storage ferrosome system in *C. difficile* to combat iron sequestration by the host in the inflamed gut during infection (82), the *fezAB CD630_05910–05920* genes are also downregulated *in vivo* in the present study (Table S2).

As observed in previous *in vivo* transcriptomics (26, 29), many sporulation genes (about 100) were induced *in vivo*, including several σ^K^-dependent genes associated with the synthesis of the outer layers of the spore (cortex, spore coat, and exosporium) (83–85) (Fig. S8A). These results confirm that sporulation is rapidly induced during infection, allowing *C. difficile* to persist in the host gut and disseminate in the environment, despite the host immune response.

Overall, our results are fully consistent with previous *in vivo* transcriptomic analyses in monoxenic or conventional mice (26, 29, 30), which perfectly validate our model.

### Differential expression of *C. difficile* ncRNAs between *in vitro* culture and infectious conditions

Among the 61 differentially expressed ncRNAs (40 induced and 21 repressed) (Table 1), several have been previously identified in a RNA-immunoprecipitation sequencing (RIP-seq) experiment as being associated with the Hfq protein (18). For example, RCd6 is repressed, while CD630_n00930 and CD630_n00620 are induced during infection (Fig. S8B). Another Hfq-associated RNA RCd5 upregulated *in vivo* is a type I riboswitch binding to c-di-GMP induced during the stationary phase of growth. Inversely, a type II riboswitch CD630_Cdi2_4 and associated *pilA* gene encoding type IV pilus component are downregulated *in vivo*. A number of *cis*-acting RNA regulatory elements related to amino acid metabolism have been identified as differentially expressed *in vivo* as compared with *in vitro* conditions. For example, 10 T-boxes responding to tRNA aminoacylation level associated with corresponding aminoacyl-tRNA synthetase or amino acid transporter genes and three lysine riboswitches upstream of lysine metabolism genes were upregulated *in vivo*, reflecting translational machinery and metabolic adaptations during infection (Table 1). Among antisense RNAs with highest differential ratio of expression between *in vitro* and *in vivo* conditions, we identified CD630_SQ1076, a putative antisense RNA of the *map2* gene encoding a methionine aminopeptidase that was upregulated during infection (Fig. S8B). The antisense RNA most highly repressed during infection was CD630_n00830, an antisense RNA of the *grdB* gene coding for a subunit of glycine reductase (Fig. S8B). Interestingly, the *grdB* gene and associated CD630_n00830 antisense RNA are inversely co-regulated *in vivo* as compared with *in vitro* conditions (Fig. S8B). Similar inverse regulation was also observed for the proline reductase operon induced *in vivo* and the antisense RNA overlapping the 3′-end expressed *in vitro* (Fig. S8B), consistent with the importance of the use of these amino acids *in vivo* for Stickland reaction. Our analysis revealed several previously uncharacterized ncRNAs as highly upregulated during infection. Among them CD630_SQ995 is located in intergenic region (IGR) between *CD630_15111* and *cotB* gene for a spore outer membrane protein, CotB (Fig. 4B), that were also induced *in vivo;* and CD630_SQ1296 is located in IGR between *CD630_18800* gene encoding ketopantoate reductase and *pyrE* gene encoding orotate phosphoribosyltransferase in the vicinity of the sequence coding for a fragment of an ABC transporter (Fig. 4B). The overexpression *in vivo* of these two previously uncharacterized ncRNAs has been validated by independent qRT-PCR analysis (Fig. S7B and C). CD630_n00640 is also induced *in vivo* and found between conjugative transposon Tn1549-like *CD630_18782* and *CD630_18780* genes. Altogether, these *in vivo* transcriptomic data represent invaluable resources for further detailed characterization of RNA-based regulatory mechanisms during CDI.

### Comparison with available *C. difficile in vivo* transcriptomic data

Several studies previously explored the *in vivo* transcriptomics of *C. difficile* in mouse model of infection, but the ncRNA genes have not been included into these analyses. We thus selected representative raw RNA-seq data sets from two independent studies (27, 32) for further comparative analysis with the present study (Table S4). This analysis revealed a total of 2,258 and 2,319 *C*. *difficile* genes differentially expressed *in vivo* as compared with *in vitro* conditions, respectively (1,180 and 1,046 genes upregulated, while 1,078 and 1,273 genes downregulated, respectively). Despite the differences in the experimental conditions and post-infection time points, *χ^2^* tests for pairwise comparisons of the three experiments revealed significant overlap for up- and downregulated genes (Fig. S9A and B; Table S5) encoding virulence factors, sporulation, stress response, and metabolism-related proteins in accordance with previous reports (26–28, 32). The functional gene-set enrichment revealed two classes associated with sporulation and lipid metabolism as upregulated in all three studies, while regulations and stress-related genes were downregulated in the present study and in Fletcher et al. or in Pruss et al.’s report, respectively (Fig. S9D and S3B). The differences observed in membrane transport and amino acid metabolism groups between the present study and two previously reported data sets could be explained by differences in experimental conditions, including later post-infection time points (2 and 3 days post-infection), different antibiotic treatment and infection mode in mouse models used and different *in vitro* culture conditions (overnight culture in TY or defined medium) in Fletcher et al. and in Pruss et al.’s reports (27, 32). Importantly, the analysis of raw sequencing data from three independent studies identified a number of ncRNA genes that were differentially expressed during infection *in vivo* as compared with *in vitro* conditions (Fig. S9B and C; Table S5). Among them, 38, 54, and 24 were upregulated and 19, 36, and 59 were downregulated in the present study and Fletcher et al. and Pruss et al.’s data sets, respectively (27, 32). Strikingly, pairwise comparison revealed a significant overlap with 22 and 13 upregulated ncRNAs in the present study as compared with Fletcher and Pruss’ data, respectively (Fig. S9B). Importantly, CD630_SQ1296 and CD630_SQ995 ncRNAs expression was highly induced, while the expression of CD630_n00830 antisense RNA was highly reduced *in vivo* in all three independent studies (Fig. S9C). This comparative analysis strengthens the results of present study identifying several ncRNAs as potential key regulators for *C. difficile* adaptation inside the host.

### Prediction of small transcripts from RNA-seq data

We then took advantage of transcriptomics data from the present study combined with raw data sets from Fletcher and Pruss (27, 32) to search for new transcripts in *C. difficile* using DETR'PROK pipeline (53). By combining the three independent data sets for *in vivo* and *in vitro* conditions, this analysis revealed 118 potential new transcript candidates in *C. difficile*, including 12 transcripts overlapping annotated CDS and 106 potential new ncRNA genes. Among them, 83 potential ncRNAs were predicted in antisense orientation to annotated genes and 23 without overlap with annotated genes (Table S6). Fifty-six of new ncRNAs were identified in antisense orientation to CDS, while 20 corresponded to antisense RNAs for previously identified ncRNAs, and seven were identified in antisense orientation to both CDS and ncRNAs. A number of these potential antisense RNAs have been detected in our Hfq RIP-seq analysis (18). Among them, an antisense RNA for *CD630_32360 prdF* proline reductase gene was repressed during infection (Fig. S8B). Interestingly, 12 antisense transcripts have been detected for riboswitches, three for CRISPR RNAs and six for sRNAs associated with Hfq RIP-seq signal. Three antisense RNAs corresponded to previously identified type I toxin–antitoxin system components (15, 16, 86) missing from current NCBI annotation. Eleven predicted RNAs corresponded to longer transcripts overlapping previously annotated ncRNAs or CDS with 3′UTR. Interestingly, in accordance with Hfq RIP-seq analysis (18) DETR’PROK pipeline detected three new ncRNAs in IGR of *CD630_22170–22180, CD630_26100*, and *CD630_33640–33650* genes and 12 additional IGR ncRNAs associated with lower signal. Altogether, these analyses contributed to the definition of the *C. difficile* transcriptomics landscape, highlighting the extent of antisense transcription, specifying the boundaries of some previously annotated transcripts, and enriching the *C. difficile* ncRNA repertoire for future studies.

### Transcriptomic analysis of host response to *C. difficile* infection

**Fig 5:**
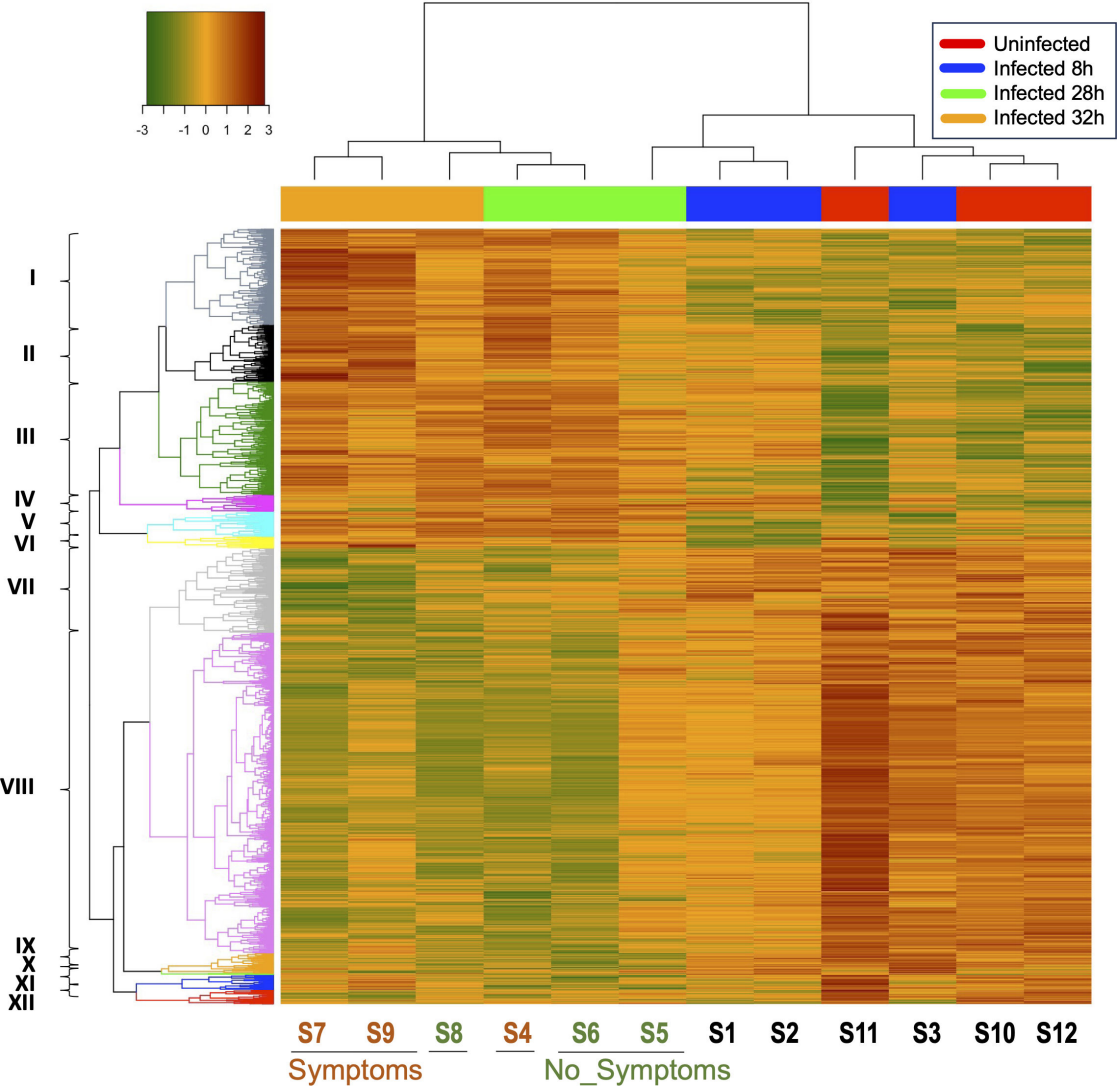
Differential gene analysis of mouse genes expression during *C. difficile* infection. A hierarchically clustered heatmap and dendrogram show the expression patterns of the genes differentially expressed in mice between uninfected control mice (Uninfected, samples S11, S12, S13), infected mice at 8 h (Infected 8 h, samples S1, S2, S3), 28 h (Infected 28 h, samples S4, S5, S6), and 32 h (Infected 32 h, samples S7, S8, S9) post-infection. Symptoms: mice infected with *C. difficile* showing visible symptoms (samples S4, S7, S9). No_Symptoms: mice infected with *C. difficile* showing no visible symptoms (samples S5, S6, S8). Clusters of genes are numbered from I to XII and discussed in the text. The color key represents the level of expression for each gene.

On the mouse side, our analysis revealed 2,297 genes significantly differentially expressed (fold change <−2 or >2, *P* < 0.05) between all conditions. Among them, 800 correspond to regulatory RNA genes, 294 induced and 506 repressed, with mostly lncRNAs (788 differentially expressed, 293 induced, and 495 repressed) and few microRNAs (12 differentially expressed, 11 induced, and one repressed). The heatmap summarizes the comparison of each mouse transcriptome (Fig. 5) and shows the expression profile of the uninfected (red), 8 h (blue), 28 h (green) and 32 h (orange) post-infection mice. Depending on their differential expression, these 2,297 genes could be grouped in 12 clusters, numbered I to XII (Fig. 5). Overall, the gene expression profiles of the 28 and 32 h infected mice (except S5 sample) are fairly similar and distinct from the 8 h infected and non-infected groups. Following this overview, we focused on main transcriptomic differences between groups.

For each gene cluster, enrichment analyses identified several differentially expressed pathways or gene families discriminating infected and uninfected mice. As expected, several specific host inflammatory markers were induced during CDI, including members of the TNFα signaling pathway (IL-1β, NLRP3, TNF, CCRL2, NFKBIA, and FOS), and chemokines (CCL4 and CCXL1) (Table S7). These inflammatory markers from clusters I and II (Fig. 5), were particularly induced in 28 and 32 h infected mice with clinical signs (diarrhea) and a highly inflamed cecum, unlike the other symptom-free 28 and 32 h infected mice. The strong overexpression of several inflammatory markers in these sick mice revealed a stronger immune response to CDI consistent with the strong cecal inflammation visually observed during animal sacrifice. This expression profile, with the induction of TNFα, IL-1β, or CCL4, reveals the activation of a T Helper type 1 (Th1) immune response, while no evidence of Th2 response was observed.

Gene clusters III, V, and VI were also induced in the 28 and 32 h infected mice both symptomatic and symptom-free. Genes from cluster III are involved in cell division and DNA repair including Nupr1, involved in regulation of cellular catabolic process or programmed cell death, already shown as part of the host response to bacterial infection (87, 88).

Few genes (cluster IV) show a distinct profile, with an induction in all infected (8, 28, and 32 h) mice. Most of these genes are part of metabolism pathways, but two could also be related to immune response, including bovine leukocyte antigen family member 2 (BOLA2), upregulated in CD4+ T cells by JAK-STAT signaling following IL-12 stimulation, and then Th1 immune response (89), and G protein subunit alpha transducin 3 (GNAT3) encoding a taste receptor, which is also expressed in the gut with potential role in innate immunity (90).

Among genes repressed during CDI (clusters VII – XII), we found many genes encoding proteins involved in (i) metabolism (fatty acid metabolism, cholesterol homeostasis, glycan, and glycosaminoglycan metabolism, …); (ii) cell junction interactions and cell adhesion molecule (cadherin, claudin, contactin, ...); (iii) signal transduction (ligand-gated ion channel as glutamate receptor, adrenergic receptor, and calcium channel) (Table S8). Similar results were obtained in dual RNA-seq experiments with *Yersinia pseudotuberculosis* proliferating in the gut-associated lymphoid tissue or *Eimeria tenella*-infected cecal tissue with shutdown of pivotal cellular functions in response to the infection (23, 91).

As no statistically significant differences in gene expression were observed with principal component analysis (Fig. S10A) between 28 and 32 h infected mice, these two groups could be combined into a late infected mice group to explore the mouse gene expression profile associated with CDI; 1,780 genes were significantly differentially expressed between late infected mice and uninfected mice (Fig. S10A and S11A) (530 repressed and 1,250 induced during infection). Among the most induced genes in infected mice were host immune response genes encoding TIR adaptor protein (TIRAP), FOS (transcription factor), NLRP3 (member of the NLRP3 inflammasome complex) and several cytokines (TNFα, IL-1α, IL-1β, IL-6, IL-22, CXCL1-2-5, CCL2-3-4-7). We validated the overexpression of IL-1β, Il-22, and CXCL5 gene in late infected mice as compared with uninfected mice by independent qRT-PCR experiment. Several genes encoding anti-microbial peptides, such as α-defensins or intelectin-1, were repressed in late infected mice (92). It has been previously observed that a parasite, *Cryptosporidium parvum*, was able to downregulate these genes as another immune evasion strategy (93).

The large number of reads aligning to the mouse genome allows for more detailed comparisons between different groups of samples. We therefore looked for potential differences based on kinetic or clinical criteria (Fig. S11B and C) by comparing the expression profile of late infected (28 and 32 h) vs early (8 h) infected mice and of symptomatic (sick) vs asymptomatic (healthy) mice within the late infected mice group.

When the combined group of 28 and 32 h late-infected mice was compared with the 8 h early infected mice group (Fig. S10B), we found a much lower number of differentially expressed genes than for the previous analysis (late infected mice versus uninfected mice). Only 375 genes were significantly differentially expressed (98 repressed and 277 induced), suggesting the quick induction of host response to CDI with the majority of genes differentially expressed as early as 8 h post-infection (e.g., inflammatory response *TNFα, IL-6*, and *IL-22*), although the histological and clinical consequences of the inflammatory process are not yet observable.

Finally, within the late infected mice (28 and 32 h), we were able to compare gene expression between sick mice vs symptom-free mice. Despite a somewhat different gene expression profile of sample S5 compared with other asymptomatic mice samples, this analysis revealed a number of potential markers for extensive inflammatory processes during CDI. We identified as many as 3,538 significantly differentially expressed genes in the sick animals, 1,909 induced and 1,629 repressed (Fig. S10C). The differentially expressed genes were classified into biological functions using Reactome (94). A large number of immune and inflammatory responses genes were induced in symptomatic mice compared with asymptomatic mice. Several highly induced genes encode proteins (calprotectin composed on S100A8 and SA1009, lactotransferrin Ltf and lipocalin 2 Lcn2) (Table 2) that contribute to nutritional immunity as an efficient antimicrobial defence strategy of the host to sequester essential divalent metals during infection (95). This induction of metal scavenging processes by the host is in line with recent studies highlighting the importance of such responses in the context of CDI (82). Other induced genes are involved mostly in metabolism, homeostasis, signal transduction, keratinization, and tissue remodeling (Table 2). Several genes encoding metalloproteases with collagenase activity, notably the *MMP8* gene, were strongly overexpressed in sick mice, in accordance with the results of Fletcher et al. (27). Degradation of collagen by these host proteins may participate to the tissue lesions but may also sustain the growth of *C. difficile* by providing proline and hydroxyproline to the bacteria.

**TABLE 2:** Host genes strongly induced in sick symptomatic mice compared with asymptomatic mice (both being late infected animals) and miRNAs induced or repressed in mice during CDI

Gene ID	Description	Category^*a*^	Fold change
Cxcl3	Chemokine (C-X-C motif) ligand 3	Immune system	163.37
Csf3	Colony-stimulating factor 3 (granulocyte)		129.61
S100a8	S100 calcium binding protein A8 (calgranulin A)		113.38
Trim10	Tripartite motif-containing 10		93.44
Cxcl2	Chemokine (C-X-C motif) ligand 2		92.80
S100a9	S100 calcium binding protein A9 (calgranulin B)		85.57
Acod1	Aconitate decarboxylase 1		85.27
Ccl3	Chemokine (C-C motif) ligand 3		75.74
Ptx3	Pentraxin-related gene		71.11
Il1f9	Interleukin 1 family, member 9		70.23
Siglecl1	Siglec family like 1		66.63
Il1a	Interleukin 1 alpha		65.39
Ltf	Lactotransferrin		55.87
Ccl4	Chemokine (C-C motif) ligand 4		51.77
Cxcr2	Chemokine (C-X-C motif) receptor 2		48.94
Mrgpra2a	MAS-related GPR, member A2A		45.73
Mrgpra2b	MAS-related GPR, member A2B		45.44
Clec4e	C-type lectin domain family 4, member e		42.25
Hcar2	Hydroxycarboxylic acid receptor 2		36.81
Tarm1	T cell-interacting, activating receptor on myeloid cells 1		35.04
Nlrp3	NLR family, pyrin domain containing 3		33.78
Hdc	Histidine decarboxylase		33.78
Cxcl5	Chemokine (C-X-C motif) ligand 5		32.76
Clec4d	C-type lectin domain family 4, member d		31.47
Lcn2	Lipocalin 2		30.13
Il1b	Interleukin 1 beta		29.65
Il1r2	Interleukin 1 receptor, type II		29.38
Osm	Ncostatin M		28.60
Il6	Interleukin 6		26.21
Il1rn	Interleukin 1 receptor antagonist		24.05
Spp1	Secreted phosphoprotein 1		21.81
Cxcl1	Chemokine (C-X-C motif) ligand 1		21.02
Scrg1	Scrapie responsive gene 1		19.20
Il13ra2	Interleukin 13 receptor, alpha 2		19.13
Tnip3	TNFAIP3 interacting protein 3		18.23
Mcemp1	Mast cell expressed membrane protein 1		18.14
Il1bos	Interleukin 1 beta, opposite strand		17.24
Serpina3k	Serine (or cysteine) peptidase inhibitor, clade A, member 3K		16.95
Ifng	Interferon gamma		16.81
Nos2	Nitric oxide synthase 2, inducible		16.44
Trim30b	Tripartite motif-containing 30B		16.00
Plaur	Plasminogen activator, urokinase receptor		15.85
Il23a	Interleukin 23, alpha subunit p19		15.53
Lilr4b	Leukocyte immunoglobulin-like receptor, subfamily B, member 4B		15.12
Ccl17	Chemokine (C-C motif) ligand 17		13.21
Plet1	Placenta-expressed transcript 1	Metabolism	28.01
Lypd3	Ly6/Plaur domain containing 3		23.20
Fgf23	Fibroblast growth factor 23		19.60
Cemip	Cell migration-inducing protein, hyaluronan binding		19.35
B3galt5	UDP-Gal:betaGlcNAc beta 1,3-galactosyltransferase, polypeptide 5		15.00
Chac1	ChaC, cation transport regulator 1		14.64
Serpina3m	Serine (or cysteine) peptidase inhibitor, clade A, member 3M		13.38
Plet1os	Placenta-expressed transcript 1, opposite strand		13.13
Prss27	Protease, serine 27		12.90
Ly6g	Lymphocyte antigen 6 complex, locus G	Hemostasis	149.50
Trem1	Triggering receptor expressed on myeloid cells 1		53.52
Gata4	GATA binding protein 4		50.63
Slc7a11	Solute carrier family 7 (cationic amino acid transporter, y + system), member 11		21.01
Sele	Selectin, endothelial cell		17.52
F10	Coagulation factor X		14.08
Prok2	Prokineticin 2	Signal transduction	149.91
Slc4a11	Solute carrier family 4, sodium bicarbonate transporter-like, member 11		60.13
Adgrf1	Adhesion G protein-coupled receptor F1		22.58
Epgn	Epithelial mitogen		14.87
Rnd1	Rho family GTPase 1		14.22
Nkx2-9	NK2 homeobox 9		12.99
Sprr2h	Small proline-rich protein 2H	Keratinization	102.04
Krt36	Keratin 36		29.88
Csta1	Cystatin A1		27.90
Tgm1	Transglutaminase 1, K polypeptide		16.31
Sprr1a	Small proline-rich protein 1A		15.92
Krt14	Keratin 14		12.73
Prss22	Protease, serine 22	Tissue remodeling	641.36
Mmp8	Matrix metallopeptidase 8		154.66
Chil1	Chitinase-like 1		33.47
Mmp10	Matrix metallopeptidase 10		30.70
Mmp3	Matrix metallopeptidase 3		26.37
Nppc	Natriuretic peptide type C	Muscle contraction	17.63
Ankrd33b	Ankyrin repeat domain 33B	Programmed cell death	13.36
MIR1938*		Induced miRNAs in infected mice (MI *vs* MC)^*b*^	8.17
MIR3109*			8.07
MIR21a			4.18
MIR1938*		Induced miRNAs in symptomatic mice (sick *vs* healthy)^*c*^	6.72
MIR6236			6.04
MIR7678			5.32
MIR3109*			3.72
MIR7672			3.36
MIR3064			1.90
MIR3069		Repressed miRNAs in infected mice (MI *vs* MC)^*b*^	−1.72
MIR1843a			−3.45
MIR1843b*			−4.17
MIR1949*			−4.55
MIR1843b*		Repressed miRNAs in mice infected at 28 h and 32 h (late *vs* early)^*d*^	−4.00
MIR1949*			−3.70
MIR682		Repressed miRNAs in symptomatic mice (sick *vs* healthy)^*c*^	−1.85
MIR99ahg			−4.17
MIR145a			−33.33

^*a*^ The functional category of host genes or miRNA differential expression class is indicated for the first gene/miRNA of the group.

^*b*^ miRNAs from the differential analysis performed between infected mice from the 28 h and 32 h groups (MI) and uninfected mice (MC).

^*c*^ miRNAs from the differential analysis performed between symptomatic mice (sick) and asymptomatic mice (healthy) from the 28 and 32 h groups.

^*d*^ miRNAs from the differential analysis performed between mice infected at 8 h (early) and mice from the 28 and 32 h (late) groups. The common miRNAs between at least two different analyses are indicated by an asterisk (*).

### Differential expression of host ncRNAs during CDI

ncRNAs can be at the crossroad of regulatory processes governing the interactions of the pathogens with their host during infection (20, 96, 97). In the present study, many lncRNAs have been identified differentially expressed during CDI, but most of them have not yet been characterized. Compared with uninfected mice, 185 ncRNAs differentially expressed, with more lncRNAs (178 genes, 96 repressed, and 82 induced) than miRNAs (seven genes, four repressed, and three induced), have been identified in infected mice. Thirty-eight ncRNAs were differentially expressed in late infected mice compared with early infected mice (20 induced and 18 repressed), with the vast majority being lncRNAs (36 genes, 20 induced, and 16 repressed) and only two repressed miRNAs. Among late infected mice, 257 ncRNAs were differentially expressed (155 induced and 102 repressed) in symptomatic mice compared to asymptomatic mice. As in the global differential analysis encompassing all conditions, almost all of these ncRNAs were lncRNAs (248 genes, 99 repressed, and 149 induced). Moreover, in this analysis, nine differentially expressed miRNAs were identified (three repressed and six induced).

Among the ncRNAs, the miRNAs have emerged as important players in host responses to bacterial pathogen infections (96, 98). We thus extracted the miRNAs differentially expressed in mice during CDI for each comparative analysis (Table 2). Of the 14 miRNAs identified, seven were repressed, and seven were induced upon CDI. Four miRNAs were found to be differentially expressed in two of the three differential analyses. The two miRNAs, miR-1843b and miR-1949, were repressed in infected mice as compared with uninfected mice but also in late infected mice as compared with early infected mice. miR-1949 has only been described as apoptosis-related miRNA in ovarian granulosa cells induced by cadmium and also as a potential inducer of bladder cancer following spinal cord injury (99, 100). miR-1938 and miR-3109 were induced in infected mice compared with uninfected mice but also in symptomatic mice compared with asymptomatic mice. These two miRNAs could be then of particular interest for further functional characterization.

Among the miRNAs repressed in mice during CDI, three (miR-145a, miR-682, and miR-99a) have already been involved in anti-inflammatory processes, whereas no role for miR-1843a and miR-3069 has been previously identified. miR-145a negatively regulates the sepsis-induced inflammatory response through modulation of NF-κB signaling (101), miR-682 has a protective effect on intestinal cells damaged during ischaemic episodes (102) and miR-99a exerts an anti-inflammatory effect when expressed in adipose tissue by inhibiting TNF-α (103).

Among the miRNAs induced in mice during CDI, two (miR-21a and miR-7678) have already been described in inflammatory response. miR-21a, one of the most highly expressed miRNAs in mammalian cells, could play a dynamic role in pro-inflammatory responses (104). miR-7678 is regulated by TNF-α and involved in controlling the inflammatory response in tissue-engineered cartilage (105). No role in inflammatory response has been shown for miR-3064 and miR-6236, which have only been described in cardiac or brain diseases (106, 107).

Our results underline the complexity of the regulatory networks of the inflammatory response during CDI and the potential role of miRNAs and lncRNAs in this process. Nevertheless, global miRNA regulation seems to favor the inflammatory process, with reduced expression of anti-inflammatory miRNAs and induction of pro-inflammatory miRNAs. Interestingly, the comparison showing the greatest differences in expression within these regulatory RNAs was between late-infected mice that were sick or asymptomatic. A more detailed analysis of these phenomena could provide a better understanding of the relationship between infection and disease in the host.

### Conclusion

*C. difficile* interacts with host and resident microbial communities inside the gut during infection. We took advantage here of the conventional mouse model of CDI mimicking the infection in humans to follow simultaneously the transcriptome dynamics of the pathogen and the host but also the kinetics of the gut microbiota composition. Such dual *in vivo* transcriptomics approach has never been applied to *C. difficile* before, and ncRNAs were not included in previous transcriptomics during CDI, although a recent paper reports a transcriptomic profiling of *C. difficile* attached to epithelial cells using an *in vitro* human gut model over 24 h (108). This study did not look at ncRNA and, importantly, the expression of several key RNA regulators could be only detected under relevant conditions *in vivo*.

From the pathogen side, our data confirmed differential expression *in vivo* as compared with *in vitro* conditions of the toxin, metabolism and sporulation genes also observed with different infection models before and identified for the first time the ncRNA expression dynamics *in vivo*. Our dual RNA-seq analysis revealed new promising candidates among ncRNAs highly induced or repressed *in vivo* that correlated with analysis of available raw RNA-seq data sets from two independent studies. Some of these ncRNAs could be related to the regulation of sporulation process in accordance with accumulating evidence for the importance of RNA-based mechanisms in the control of this key step in *C. difficile* infection cycle including Hfq (17, 109) and Hfq-binding ncRNAs (18, 110, 111).

From the host side, our transcriptomics revealed various inflammation-related pathways as highly induced during infection. A number of known pro-inflammatory miRNAs or previously uncharacterized miRNAs and lncRNAs have been identified as differentially expressed during CDI paving the way for further functional studies of these RNA-based mechanisms modulating host responses. We identified a particular expression pattern for *C. difficile-*infected mice presenting symptoms as compared with infected but asymptomatic mice, leading to identification of promising markers associated with extensive inflammatory processes. Unfortunately, the relatively low number of *C. difficile* reads in *in vivo* samples did not allow a detailed comparison of the gene expression profiles between asymptomatic and sick late infected mice. However, the host changes between these two groups correlated with specific modifications of microbiota profiles revealing interesting candidate species that may be involved in the modulation of the inflammatory process during CDI as potential targets for further microbiota-related modulatory strategies to improve the efficiency of CDI treatments.

The present study made it possible to follow the complex interactions between *C. difficile* and its host during infection, illustrating the battle for essential nutrients including metals with strong metal scavenging processes induced in the host to combat the pathogen and specific metal transport deregulations in *C. difficile*. The pathogen secretes the toxins inducing host inflammatory responses leading to tissue lesions and successful infection, while the host induces a number of defense mechanisms producing antimicrobial peptides and activating immune responses. Both interacting organisms are experiencing profound metabolic adaptations with *C. difficile*, inducing translational activity and adjusting its metabolism to available resources in the gut in competition with resident microbiota, while the host largely shuts down its metabolism and other general cell functions. As an example, the induction of collagenase expression in the host contributes to tissue lesions providing resources to sustain the *C. difficile* growth, the pathogen inducing proline reductase pathway to use proline as energy source available from collagen degradation. During infection, the induction of *C. difficile* spore formation appears also as an efficient strategy to persist in the gut evading the host immune responses. Future studies will complete this first transcriptomic picture with more detailed view on gene expression dynamics and regulations during *C. difficile* interactions with its host expanding it to clinically relevant epidemic strains.

Overall, the data generated during this work represent a unique resource for scientific community to explore both the pathogen and the host gene expression during infection and show the power of combined computational approaches applied to complex data sets to extract valuable and statistically significant information on host and pathogen transcriptome, microbiome, and ncRNA identification despite the small sample size. These data constitute the essential basis to specify the RNA-based mechanisms shaping virulence and adaptation of *C. difficile* to its host and modulating the immune and inflammatory host responses. By identifying specific virulence markers and potential therapeutic targets, this work opens new avenues for future development of alternative therapeutic and diagnostic strategies.

## Data Availability

Raw sequencing data have been submitted to ENA with the accession number PRJEB64651.

## SUPPLEMENTAL MATERIAL

Supplemental Figures msystems.00863-24-s0001.pdf Figures S1-S11.

Supplemental Text msystems.00863-24-s0002.pdf Supplemental methods.

Supplemental Tables msystems.00863-24-s0003.pdf Tables S1, S3, S4, S7, and S8.

Table S2 msystems.00863-24-s0004.xlsx Differential expression analysis of *C. difficile* genes.

Table S5 msystems.00863-24-s0005.xlsx Comparison with available *C. difficile in vivo* transcriptomic data.

Table S6 msystems.00863-24-s0006.xlsx Prediction of new transcripts from RNA-seq data with DETR'PROK.

ASM does not own the copyrights to Supplemental Material that may be linked to, or accessed through, an article. The authors have granted ASM a non-exclusive, world-wide license to publish the Supplemental Material files. Please contact the corresponding author directly for reuse.
