# Supplementary material for: Dual RNA-seq study of the dynamics of coding and non-coding RNA expression during Clostridioides difficile infection in a mouse model
Source: mSystems. 2024 Nov 27;9(12):e00863-24. doi: 10.1128/msystems.00863-24 (PMC11651100; doi:10.1128/msystems.00863-24)

Figure S1. Clinical signs of sickness indicating *C. difficile* infection for mice during clinical follow-up assay. Mice were evaluated for stool characteristics, behavior change and weight loss. Each parameter was scored from 0 (formed stool, normal behavior and no change in weight) to 4 (inability to deambulate, mucous stool and weight loss > 15%). Individuals scores were combined to a cumulative clinical sickness score (CSS) ranging from 0 to 12.

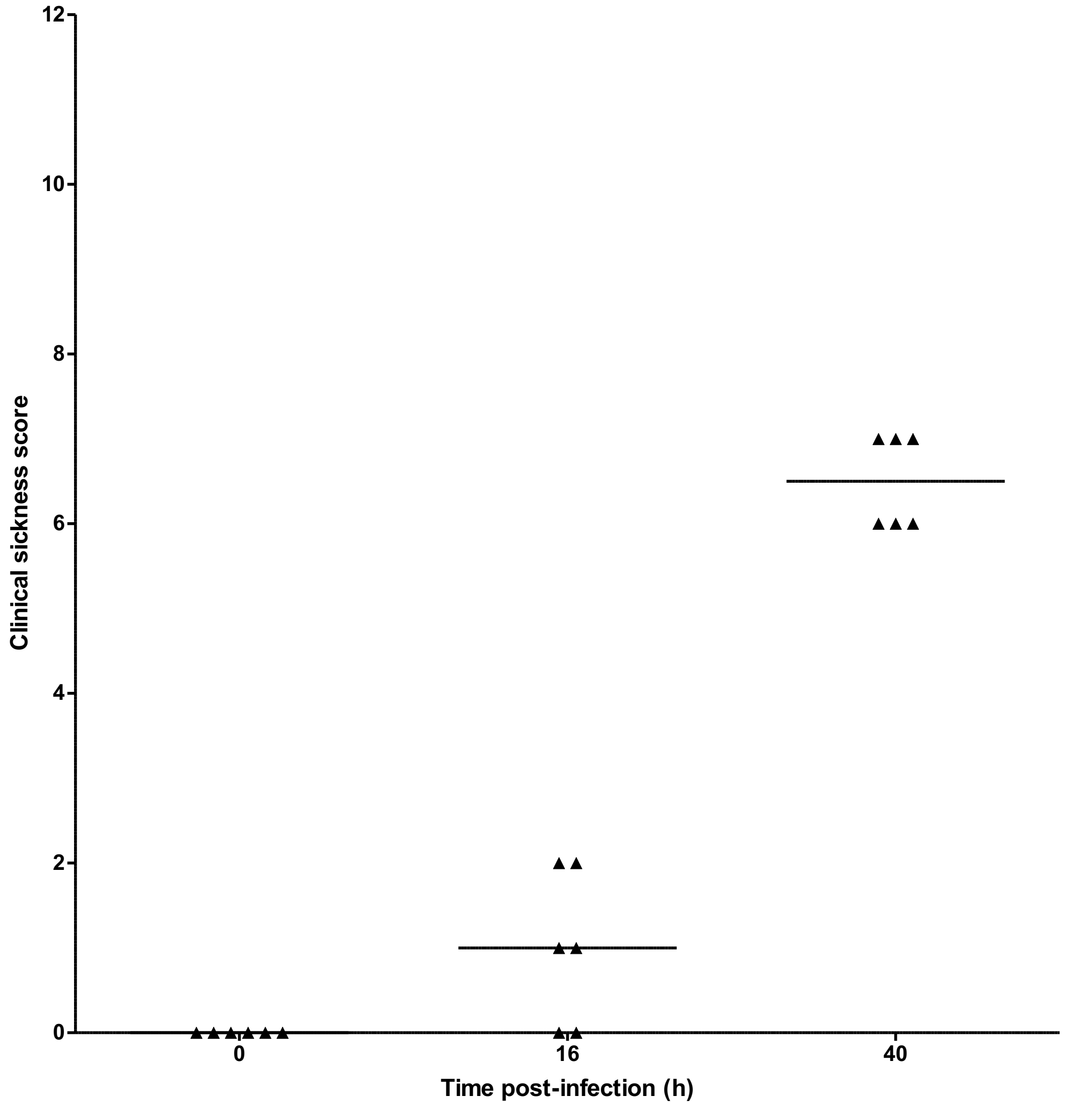

Figure S2. Mapping of dual RNA-seq reads against reference genomes of *C. difficile* and mouse and remaining reads of microbiota species in mouse samples. The total number of read counts mapped on *C. difficile* (**A**) and mouse (**B**) genome, as well as remaining read counts for microbiota species **(C)** are shown for each *in vivo* sample from S1 to S9 for infected mice 8h (in green), 28h (in yellow) and 32h (in red) post-infection as compared to *in vitro* culture samples from IV1 to IV3 (in blue in (**A**)) or uninfected mice samples S10-S12 (in blue in (**B**) and (**C**)).

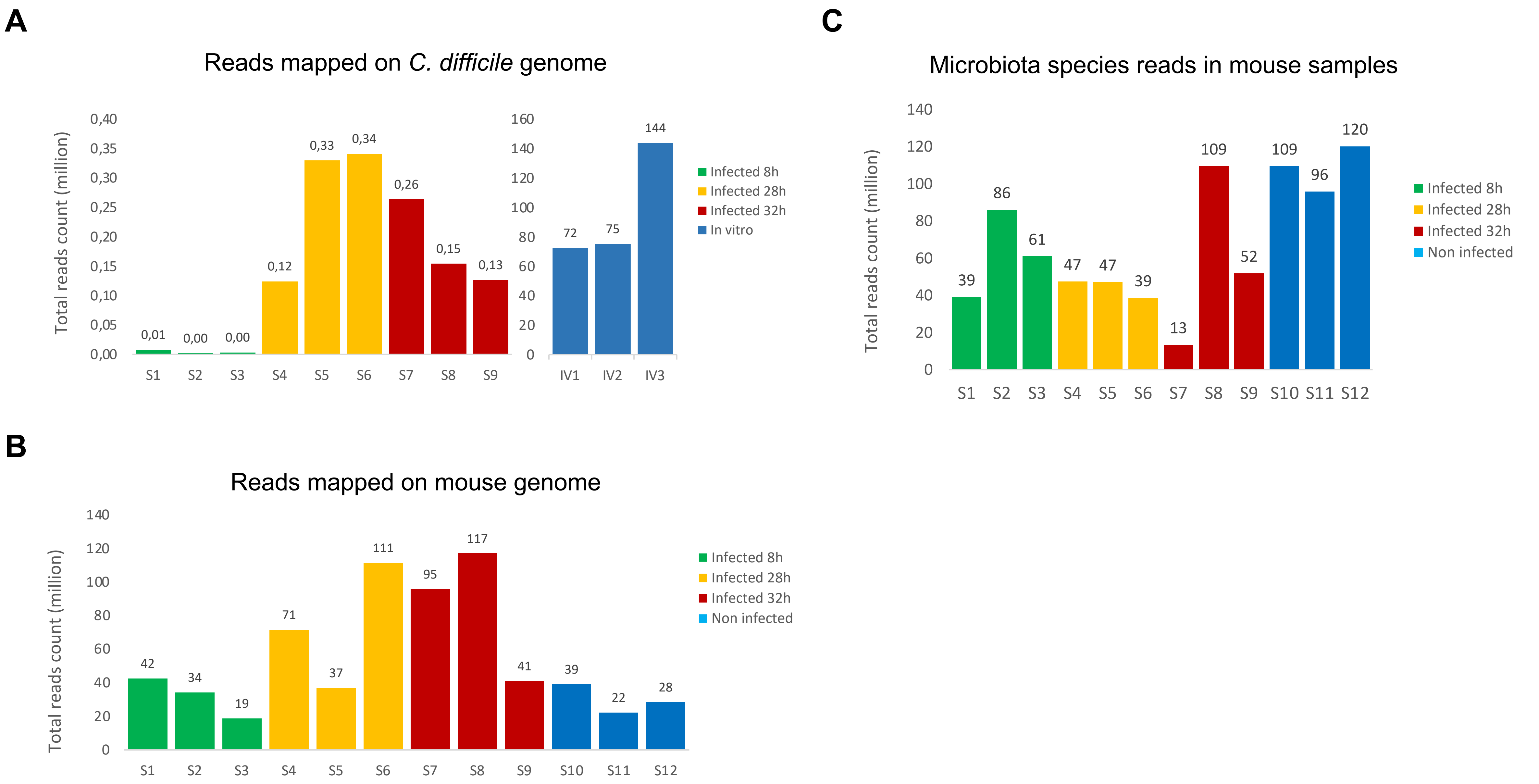

Figure S3. Principal component analysis of the differential expression analysis of *C. difficile* genes between groups IV (*in vitro*) and MI (mice infected). Each *in vivo* sample from S4 to S9 for late-infected mice 28h and 32h post-infection and *in vitro* culture samples (IV) is represented. The PC1 axis allows to separate the samples from the different conditions compared (*in vivo vs in vitro*), meaning that biological variability is the main source of variation in the data analyzed.

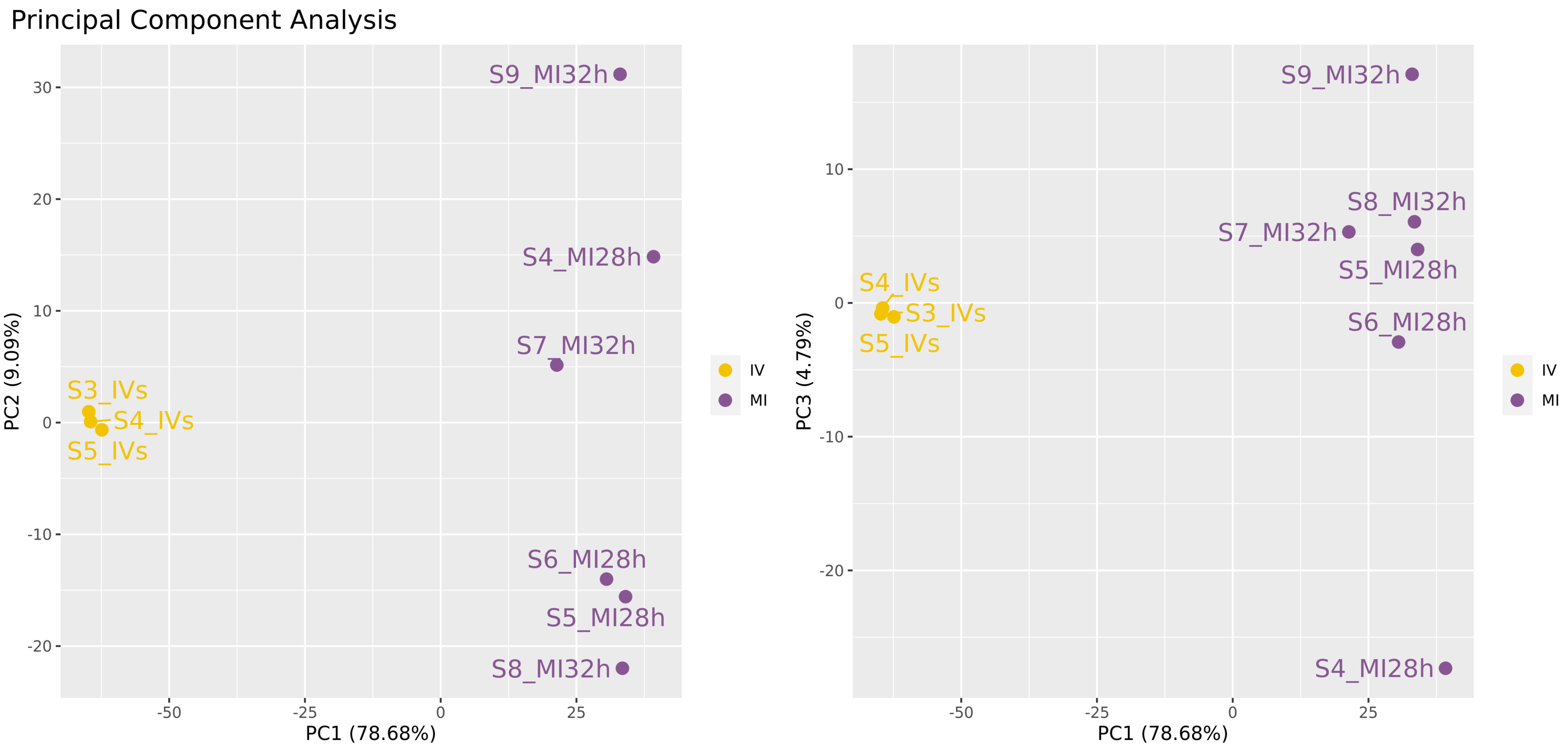

Figure S4. Analysis of microbiota species identified in the mouse gut. (A) Two-dimensional PCA loading plot. Arrows and text represent the loadings (species) with their contribution on sample separation. **(B) Heatmap of clustering based on species relative abundance across the different samples.** Row scaling was done by applying the unit variance scaling and the clustering of both rows and columns was performed using correlation distance and average linkage. Scale of relative distances is represented on top right ranging from 2 to -2. NOT_INF: mice not infected with *C. difficile* (samples S10, S11, S12), SYMPTOMS: mice infected with *C. difficile* showing visible symptoms (samples S4, S7, S9), NO_SYMPTOMS: mice infected with *C. difficile* showing no visible symptoms (samples S5, S6, S8).

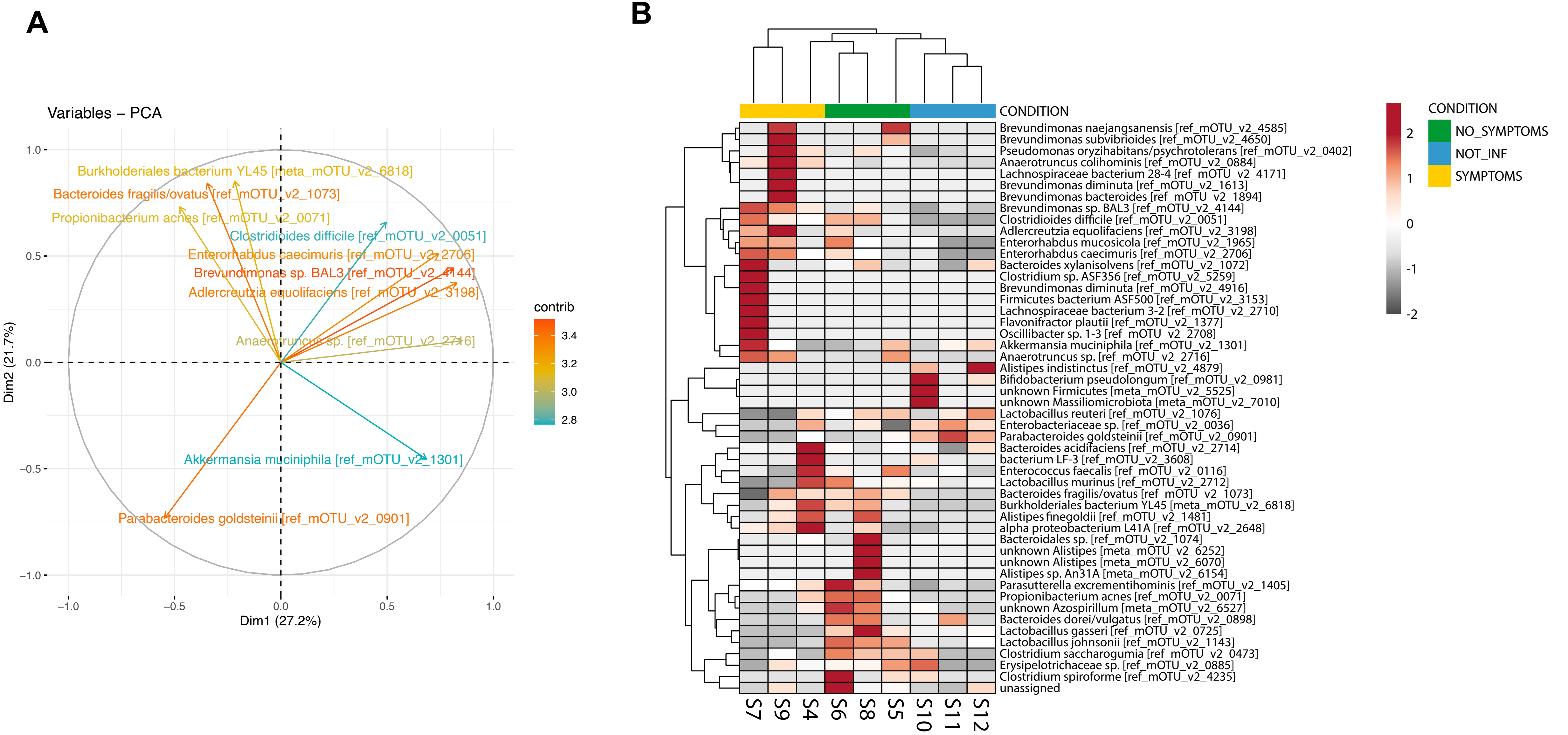

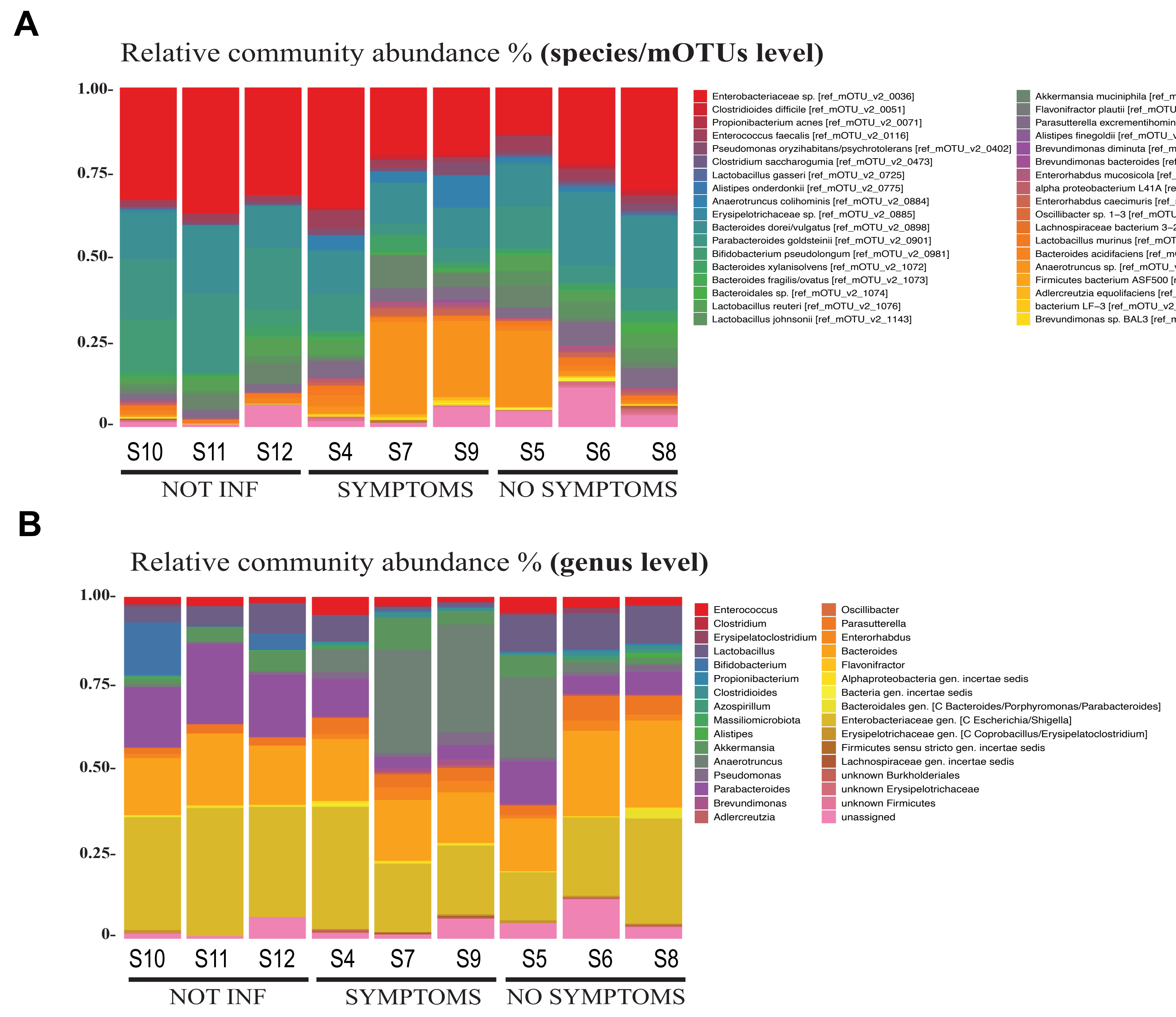

Figure S5. Relative community composition of the mouse gut microbiota at the species/mOUTs (A) and genus (B) level determined by metatranscriptomics profiling on housekeeping marker genes using mOTUs2. The experimental conditions are shown under the stacked bars: NOT_INF : mice not infected with *C. difficile* (samples S10-S12). SYMPTOMS : mice infected with *C. difficile* showing visible symptoms (samples S4, S7, S9). NO_SYMPTOMS : mice infected with *C. difficile* showing no visible symptoms (samples S5, S6, S8).

Figure S6. Functional GSEA analysis of *C. difficile* genes differentially expressed in mice during infection as compared to *in vitro* conditions. (A) running sum of gsea analysis for Sporulation (left) and Regulations (right) classes; **(B)** proportion of *C. difficile* leading genes given by the GSEA analysis with the MA2HTML categories, up (left) and down (right) regulated genes in the mice infected *versus in vitro* growth conditions.

A

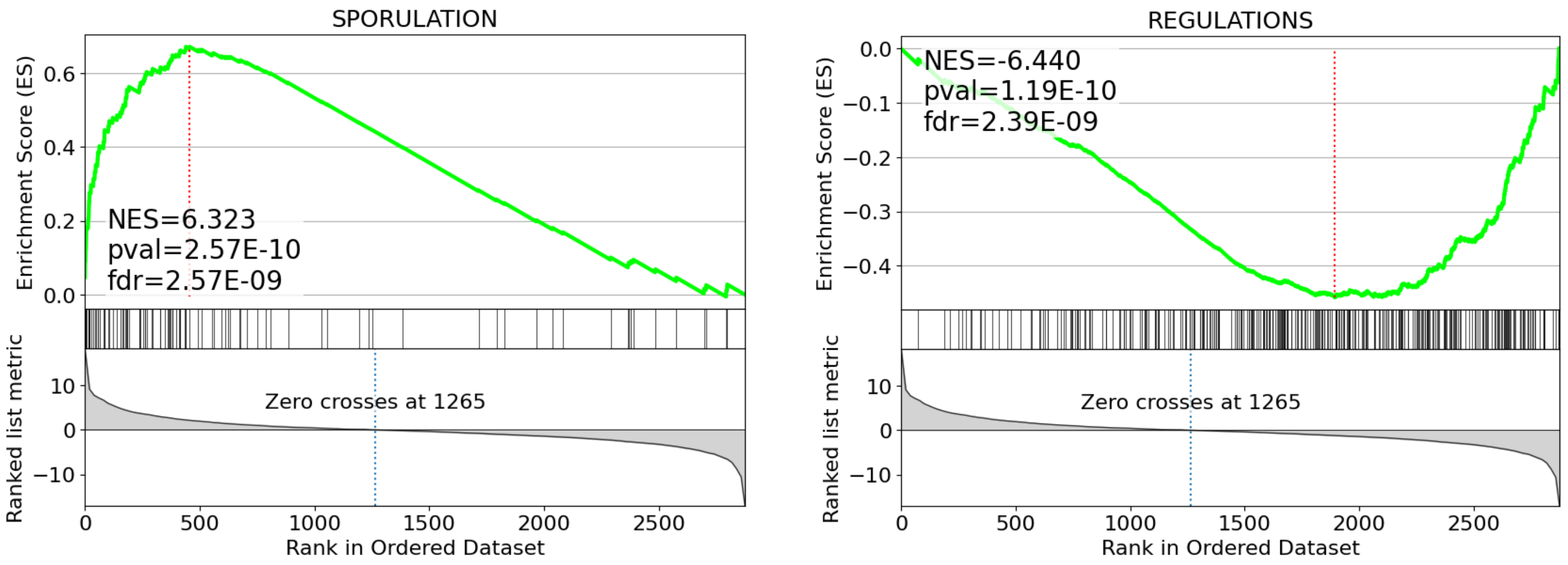

B

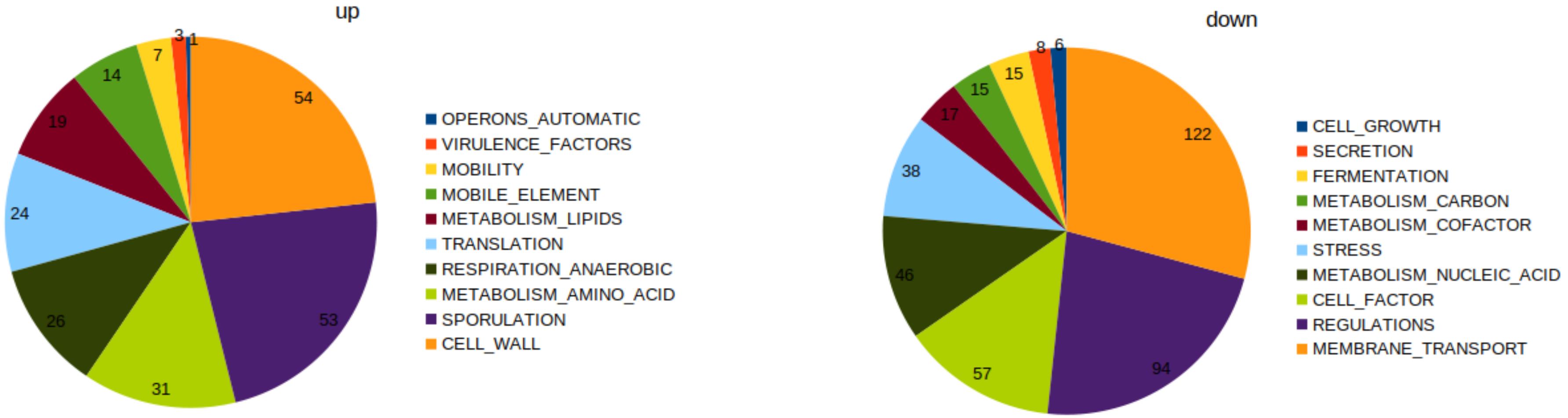

Figure S7. Real time qRT-PCR of *in vitro*, 28h post-infection (left) and 32h post-infection (right) samples, on *tcdA* (A), and ncRNA SQ995 (B) and SQ1296 (C) genes. Not infected mice have been used as control. The asterisk indicates a statistically significant difference (* $p<0.05$, ** $p<0.01$, ***$p<0.001$, ***$p<0.0001$). Data are the mean (±SEM) of at least three independent assays.

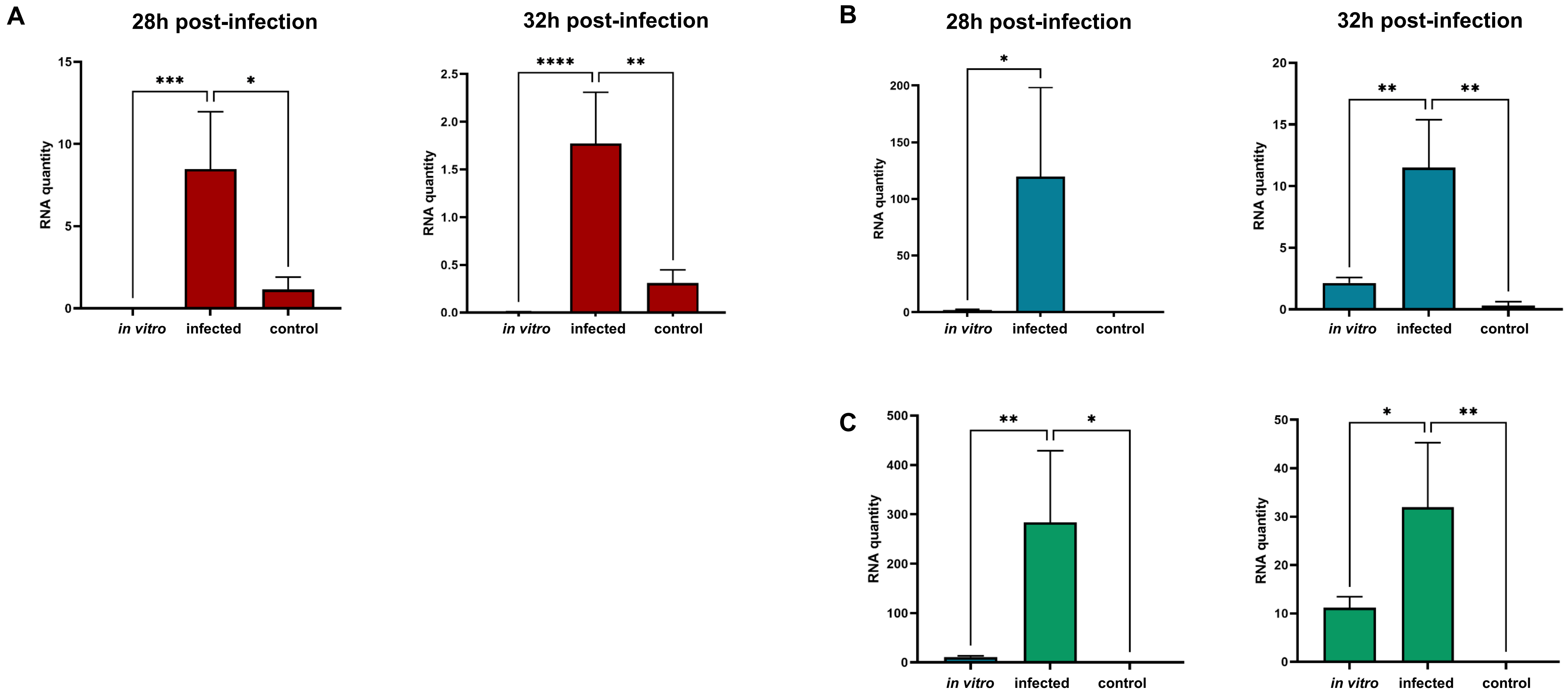

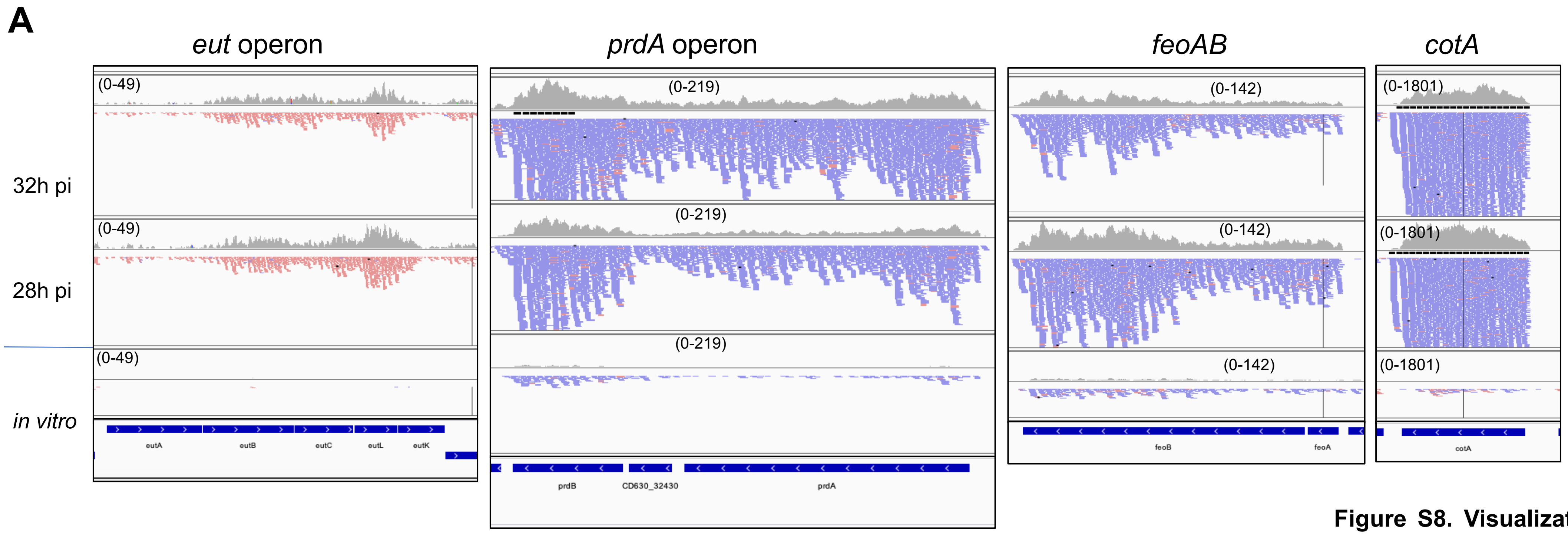

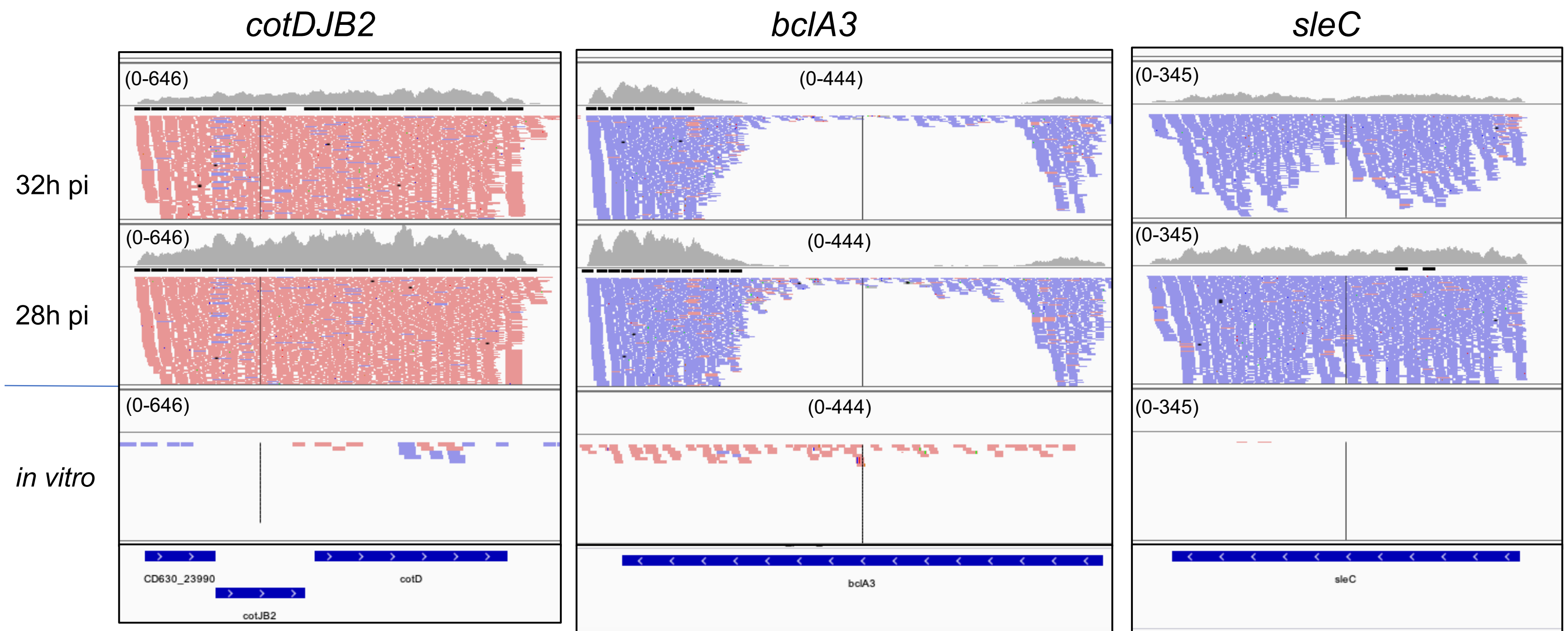

Figure S8. Visualization of dual RNA-seq data for *C. difficile* differentially expressed genes with IGV. Representative examples from different functional groups of genes are presented in **(A)** for protein encoding genes including sporulation proteins, in **(B)** for ncRNAs. The results for *in vivo* samples 28h post-infection (28 pi), 32h post-infection (32 pi) are compared with the data from *in vitro* sample. The genomic regions for previously identified ncRNAs are presented in a green box, newly identified transcribed region are shown in orange box. Genes from MaGe annotation are shown at the bottom of each panel. In the IGV visualization profiles, "+" strand reads are shown in red and "-" strand reads are shown in blue. IGV visualization is presented with adjusted read threshold for each window to compare the data from different samples (scale is indicated as a read threshold range).

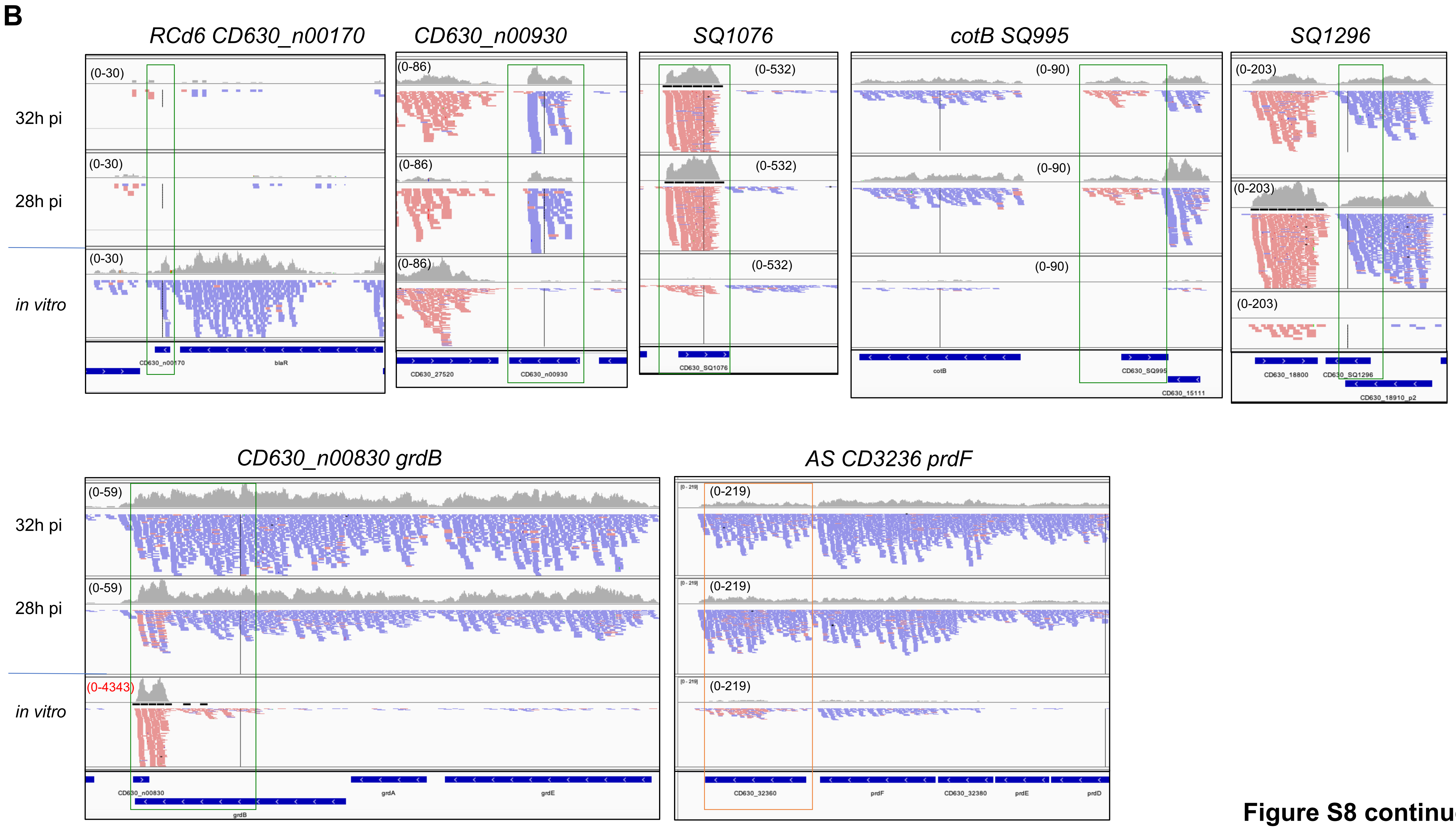

Figure S8 continued

Figure S9. Comparison with available *C. difficile in vivo* transcriptomic data.
(A) Venn diagram showing the number of differentially expressed genes that are up (left) and down (right) regulated *in vivo* in the infected mice versus *in vitro* growth conditions in this study (in red) as compared to previously reported datasets in Fletcher *et al.* (in blue) [27] and in Pruss *et al*. (in green) [32]. **(B)** $\chi 2$ tests of pairwise comparisons of the 3 gene expression experiments in *C. difficile*. The table indicates the *p*-value for ncRNAs or CDS up- and downregulated *in vivo* as compared to *in vitro* conditions (up in top right half in red, down in bottom left half in blue). Experiments were compared pairwise. A low *p*-value indicates a dependency of differential expression up or down status between the experiments. **(C)** Heatmap of the differentially expressed ncRNAs *in vivo* as compared to *in vitro* conditions in present study "Kreis" and previous reports "Fletcher" [27] and "Pruss" [32]. **(D)** Heatmap of the differentially expressed CDS genes *in vivo* as compared to *in vitro* conditions in present study "Kreis" and previous reports "Fletcher" [27] and "Pruss" [32]. On the right is shown the enrichment analysis of Ma2HTML classes with *C. difficile* expression profiles in infected mice versus *in vitro* growth conditions for "Fletcher" [27] and "Pruss" [32] studies to compare with this analysis for present study in Figure 3B. The enrichment score reflects the concentration on one side of the genes belonging to the class (left side, red for upregulated differentially expressed genes; right side, blue for downregulated differentially expressed genes) as the genes are ordered according to their decreasing Log2FC (grey curve at the bottom). NES: normalized enrichment score; SET: class name; *p*-val : adjusted *p*-value.

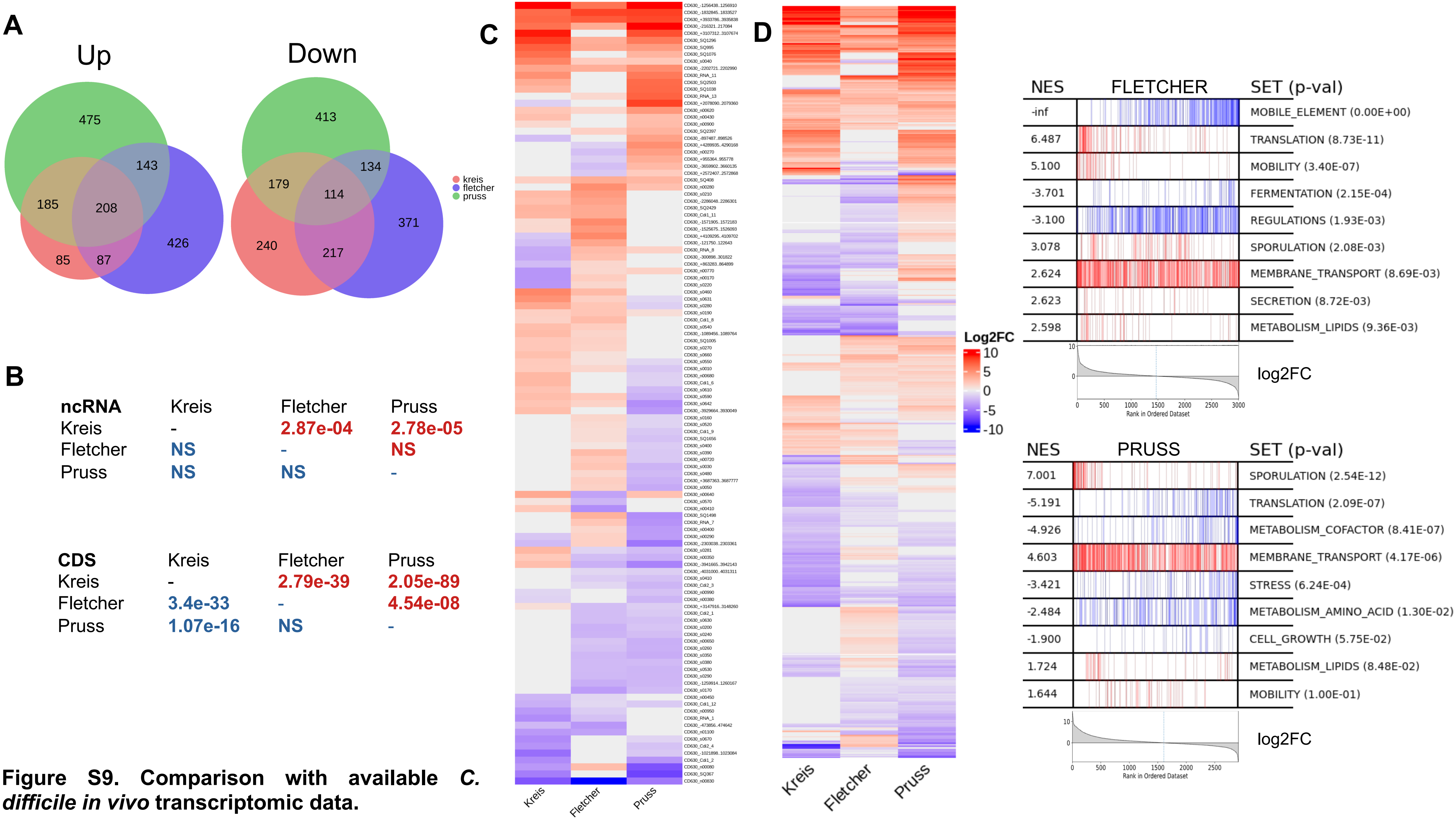

Figure S9. Comparison with available *C. difficile in vivo* transcriptomic data.

Figure S10. First two components of a Principal Component Analysis with percentages of variance associated with each axis. Comparison was made between uninfected mice (MC) and late infected mice at 28h-32h post-infection (MI) in (**A**); between early-infected mice (8h) and late-infected mice 28h-32h post-infection (28h-32h) in (**B**) and between symptomatic (sick) and asymptomatic (healthy) mice from the two late infected groups at 28h and 32h post-infection in (**C**).

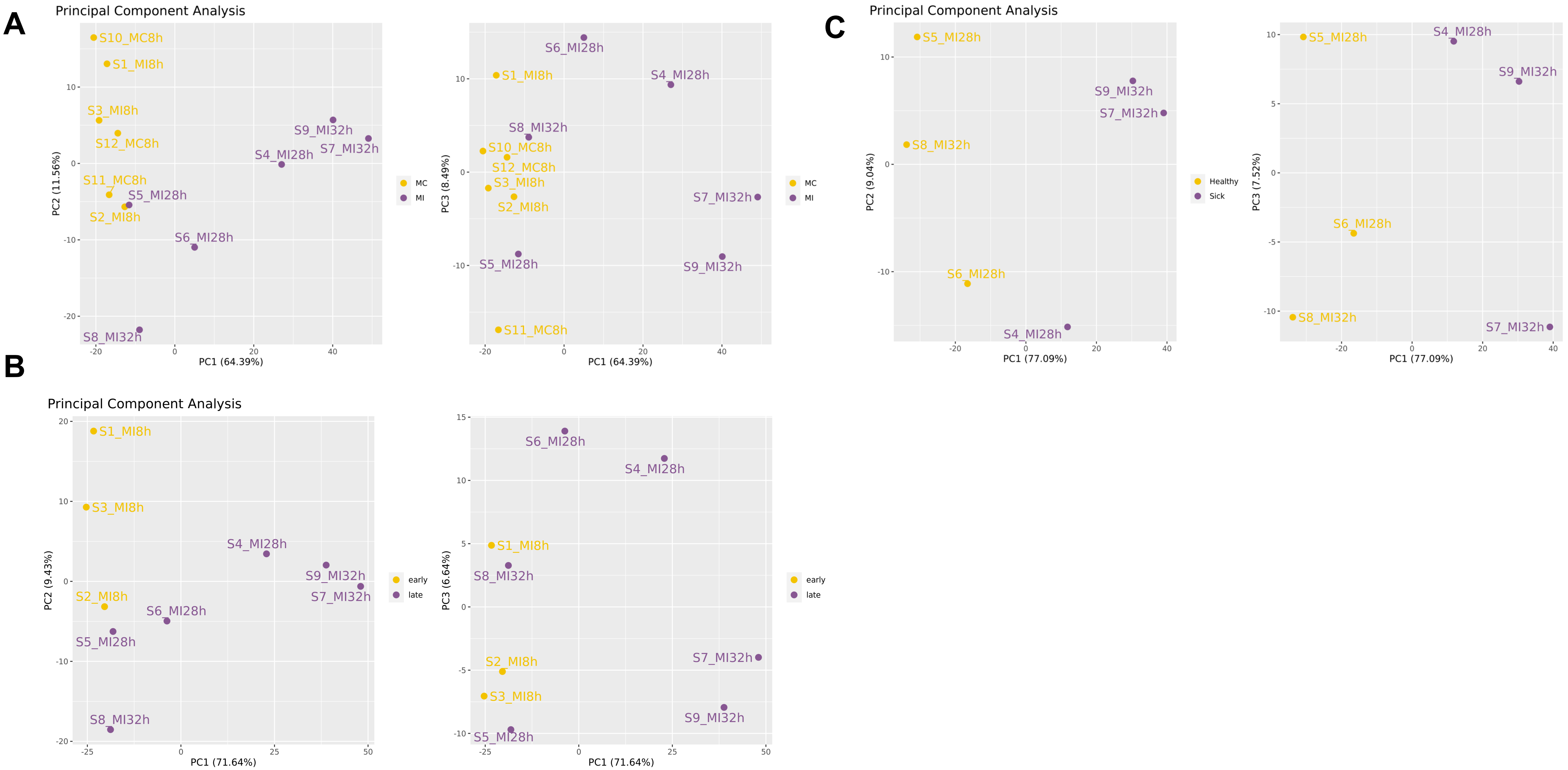

Figure S11. Differential Gene Analysis of mouse genes during infection between different groups. (A) uninfected control mice (MC) were compared to infected mice at 28h and 32h post-infection (MI); **(B)** mice infected at 8h post-infection (early) were compared to mice infected at 28h and 32h post-infection (late); **(C)** symptomatic (sick) mice were compared with asymptomatic (healthy) mice at 28h and 32h post-infection.

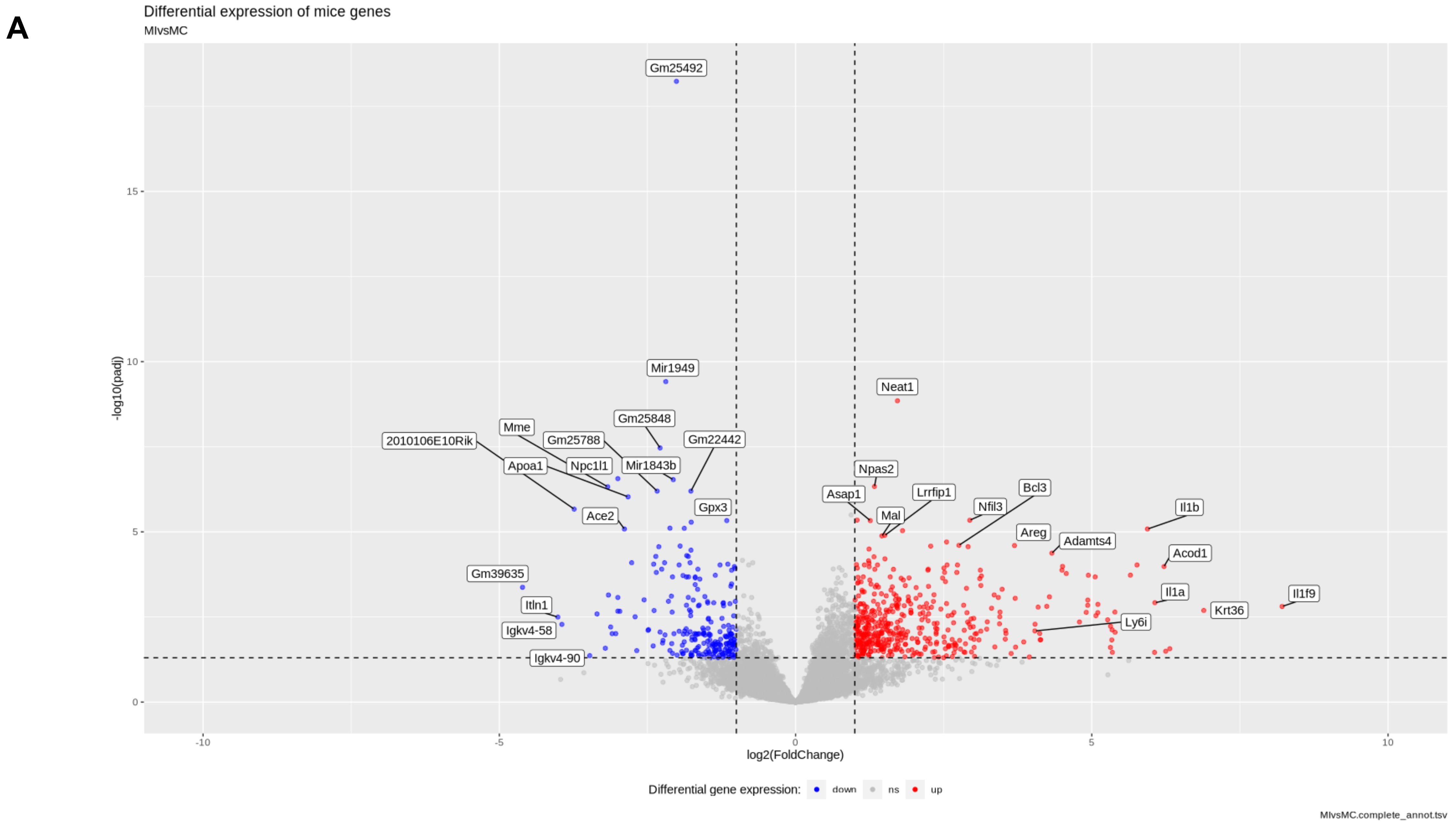

Figure S11. Continued

B

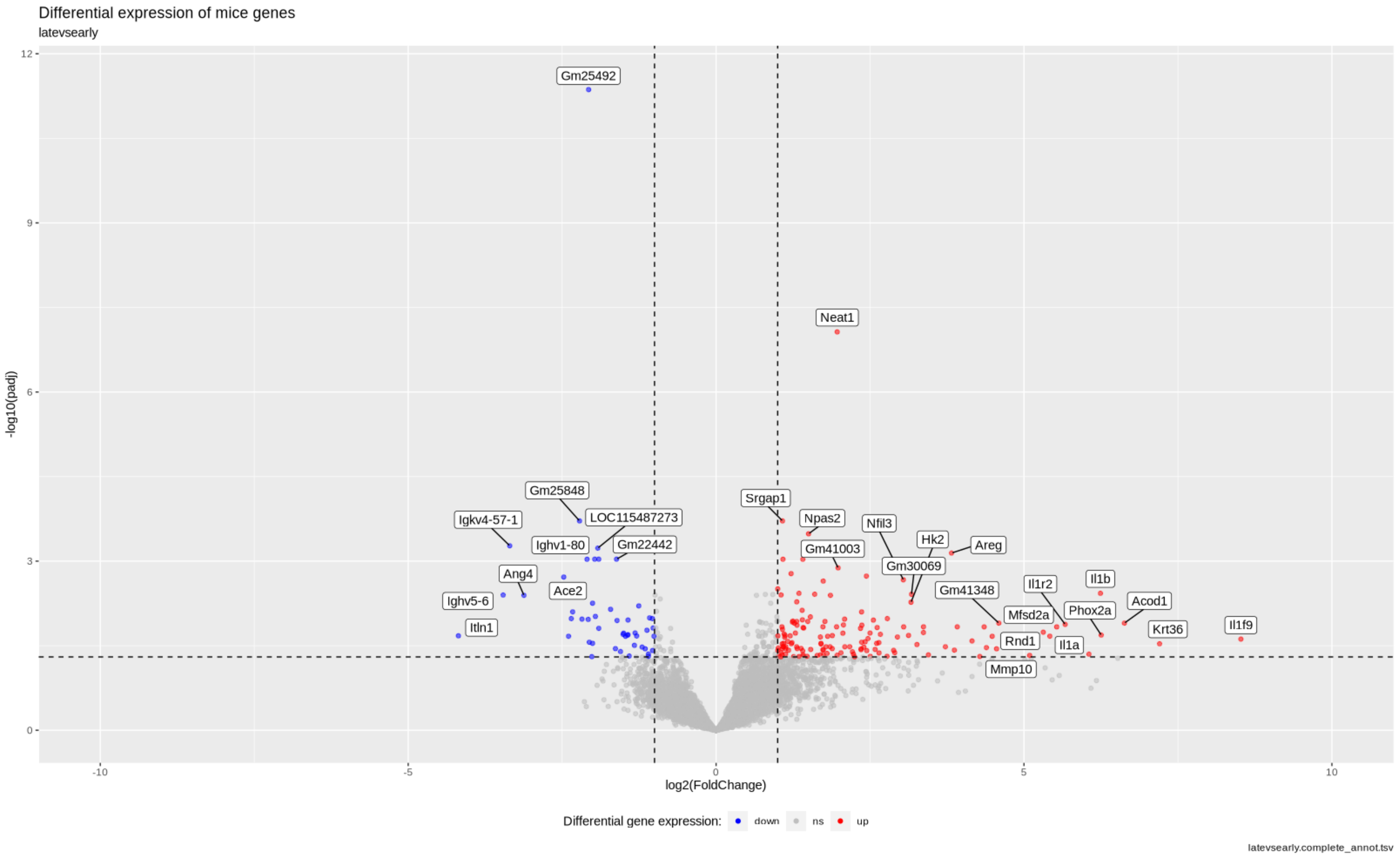

Figure S11. Continued

C

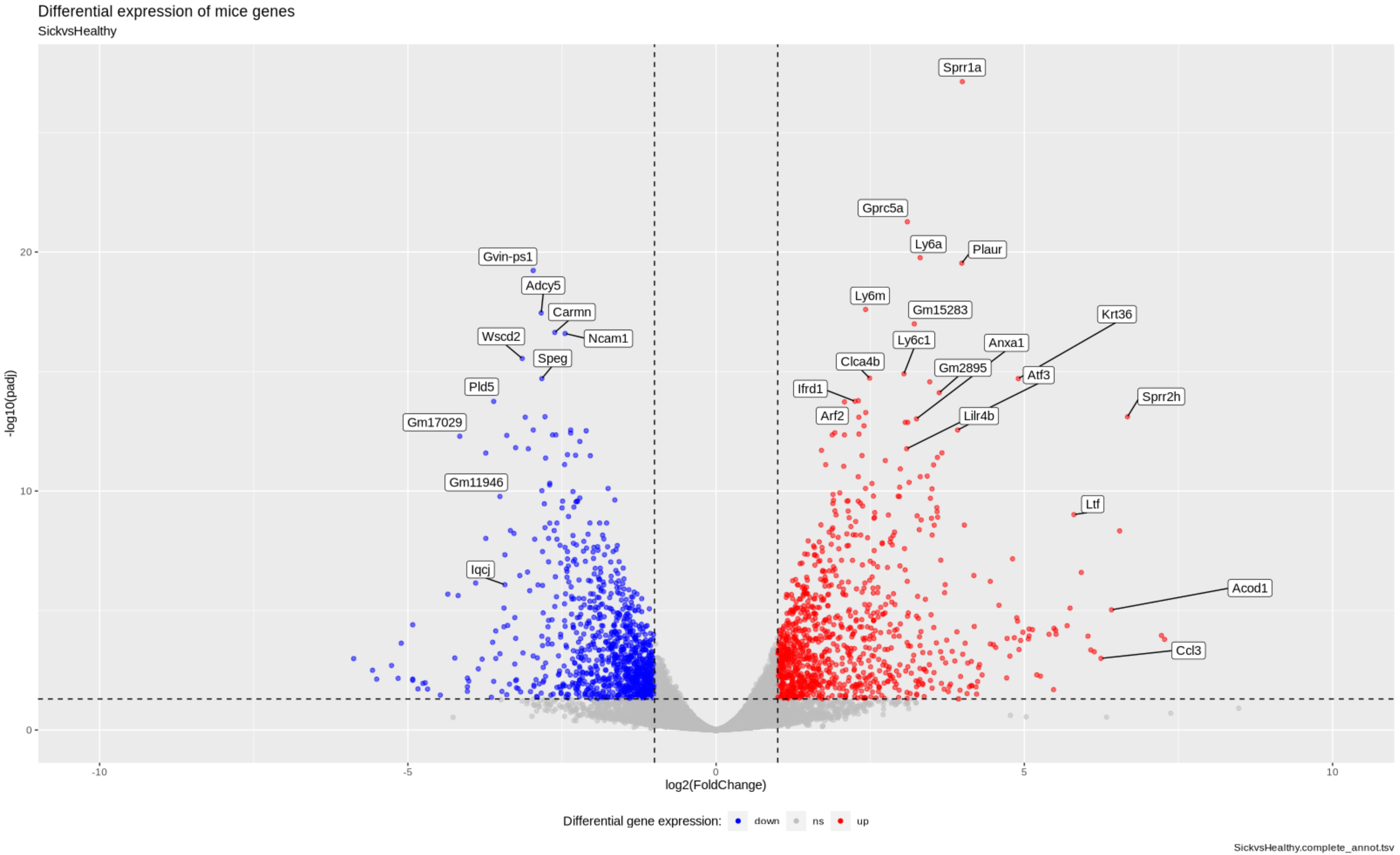

Supplement: Supplemental Figures — Figures S1-S11. [file msystems.00863-24-s0001.pdf]
