# Supplementary material for: Dual RNA-seq study of the dynamics of coding and non-coding RNA expression during Clostridioides difficile infection in a mouse model
Source: mSystems. 2024 Nov 27;9(12):e00863-24. doi: 10.1128/msystems.00863-24 (PMC11651100; doi:10.1128/msystems.00863-24)
Supplement: Supplemental Text — Supplemental methods. [file msystems.00863-24-s0002.pdf]

Supplementary methods

Animal model and treatment

An antibiotic mixture of kanamycin (0.4 mg/ml), gentamicin (0.035 mg/ml), colistin (850 U/ml), metronidazole (0.215 mg/ml) and vancomycin (0.045 mg/ml) was administered to mice in the drinking water from day -6 to day -3 before infection. Mice were further switched to autoclaved water, and then received, 24h prior to the challenge, a single dose of clindamycin (10 mg/kg) by intraperitoneal injection (IP) (Figure 1A). Mice were then randomized into 4 groups of 3 mice (1 group of control mock mice and 3 groups for CDI) and were co-housed by treatment group for the remainder of the assay.

Following sacrifice, entire *caeca* were quickly collected and opened, then aliquots of caecal content were sampled for the prompt quantification of vegetative luminal bacteria by the serial dilution method to determine the burden of *C. difficile* in each caecal content. The rest of the *caeca* and their contents were immediately placed in 10 mL of RNA*protect* solution (Qiagen) to minimize RNA degradation and changes in the gene expression level and washed by gently mixing the tissue in the RNA*protect* solution to separate the bacteria-containing caecal content and the caecal mucosa. Washed tissues were then removed and cut in half for optimal lysis and then placed in 500 µL of acidic phenol and 500 µL of aqueous solution (1/2 volume of 20% glucose and 1/2 volume of Tris 25 mM pH 7.6 EDTA 10 mM) in a FastPrep tube (MP Biomedicals).

For each mouse, the caecal content collected in RNA*protect* was sedimented in ice for 30 min and centrifuged at 300xg to get rid of fecal matter. The supernatant was then centrifuged, and the bacterial pellet was resuspended in 500 µL of the same aqueous solution as used for caecal tissues, and half of this suspension (250 µL) per tube was then mixed with 500 µL of acidic phenol in a FastPrep tube.

Clinical follow-up during preliminary assay.

During the clinical follow-up assay on 6 conventional mice infected with *C. difficile* 630Δ*erm*, animals were evaluated for stool characteristics, behaviour change and weight loss in order to establish a clinical sickness score (CSS) indicating *C. difficile* infection. Briefly, each of these 3 parameters (stool characteristics, behaviour change and weight loss) was scored from 0 (formed stool, normal behaviour and no change in weight) to 4 (inability to deambulate, mucous stool and weight loss > 15%). Individuals scores were combined to a cumulative score ranging from 0 to 12 [1].

***In silico* data analysis**

All codes used during this study are available on github [https://github.com/i2bc/Dual_Seq_Cdiff_Mouse].

For sequencing data processing, the FASTQ files were generated and demultiplexed using the bcl2fastq2-2.18.12 Conversion Software (Illumina). Adapters were removed using Cutadapt v3.1 and low-quality reads were removed using trimmomatic v0.39 [2] with a sliding window of 4 nucleotides, a minimum base quality score of 20, and a minimum average read quality of 25. Reads less than 10 bases in length (and their paired read) were also discarded. Residual rRNA were also depleted using sortmerna v4.2.0 with the database smr_v4.3_default_db.fasta [3]. The filtered reads were then mapped to a merged genome of *C. difficile* 630Δ*erm* (RefSeq accession GCF_000009205.2) and mouse genome (RefSeqs accession GCF_000001635.27) using Hisat2 [4] with default settings. PE reads that failed to align concordantly were recovered and mapped on the same merged genomes with Bowtie2 [5] with --omit-sec-seq option. PE reads that failed to align concordantly after bowtie 2 mapping were recovered and kept for further microbiota analysis. A 0.5% subsampling of *in vitro* samples' reads was performed with

Samtools view v1.13 subsampling shorthand option [6]. Reads counting was then performed for each species with featureCounts v2.0.1 [7] on the BAM file obtained with Hisat2 and Bowtie2. Counting matrix of Hisat2 and Bowtie2 were then merged for the two separated organisms.
Differential gene expression analysis for *C. difficile* data is carried out using the SARTools pipeline, an R pipeline for differential analysis of RNA-Seq count data [10]. We use the SARTools template script (version 1.7.3 for computing cluster) based on the DESeq2 package to analyse two biological conditions: three samples from *in vitro* (IV) culture (3 samples) versus six samples from *C. difficile* infection of mice (MI) 28 hours (3 samples) or 32 hours (3 samples) post-infection. The R SARTools pipeline provides systematic quality controls of the dataset as well as diagnostic plots (SERE statistics, hierarchical clustering and PCA). In the comparison, 314 genes (7.4%) with null read counts in the 9 samples are not taken into account for the analysis. The SERE statistic is used as an index of similarity between samples, and the values for sample pairs range from 1.17 to 3.98 for intra-condition samples and from 5.58 to 8.07 for inter-condition samples (the higher the SERE value, the lower the similarity; a value of 1 or >1 represents technical and biological replicates, respectively).
A hierarchical clustering of all samples is performed after a transformation of the raw count data (under a variance stabilization transformation to make the data homoscedastic) and the resulting dendrogram (obtained from a Euclidean distance calculated between samples, and built on Ward's criterion) groups the replicates and separates the biological conditions as attempted by the experimental design. The variability of the experiment visualized with the first principal components of the PCA separates the samples of the two conditions with a percentage of variance associated with the first axis of 78.68%.
Next, the SARTools pipeline performs DESeq2 statistical analysis on the count data: definition of scaling factors, detection of outliers, estimation of data dispersion, model fitting, hypothesis testing and *p*-value adjustment for multiple testing.
DESeq2 computes a scaling factor using the median of ratios method for each sample to get normalized read counts; scaling factors have a mean of 1.24 (median 1.3).
Outlier detection is defined following a large Cook's distance, and these genes are excluded from differential expression tests. On the basis of the negative binomial distribution of the DESeq2 model, data dispersion estimation is carried out by applying a generalized linear model (with the DESEq2 fitType option set to “parametric”). DESeq2 imposes a Cox Reid-adjusted profile likelihood maximization and uses the maximum a posteriori of the dispersion. DESeq2 performs a Wald' statistical testing and the raw *p*-value distribution obtained for the IV vs. MI comparison presents the expected L-shape. Next, DESeq2 performs independent threshold-based filtering on the mean of normalized counts; the threshold sets at 0.89, and eliminates 1124 genes out of 4242. Finally, a Benjamini-Hochberg adjustment of the *p*-value is performed to account for multiple testing with a controlled false-positive rate of 0.05, yielding 1309 differentially expressed genes (559 up- and 750 down regulated).
The pipeline for comparative analysis with available *in vivo* transcriptomics data includes quality control (fastqc [8]), genome indexing of *C. difficile* 630, read mapping (bowtie2 [5]), selection of mapped reads (samtools [6]), counting (featureCounts from the subread package [7]) based on coding sequences listed in genome annotation and supplemented with the list of ncRNAs from our previous analysis [9] and new transcript candidates (this work, see below), and differential gene expression analysis (SARTools [10]).
For prediction of new ncRNAs using DETR'PROK mapping results from all replicates of a condition were combined, followed by a sampling step (samtools view [6], subsample option) so that the resulting file size was around 10 M maps per condition (subsample 0.2 for the fwtTY condition and 0.1 for the pBase condition). To handle paired-end and oriented sequencing with the DETR'PROK pipeline dedicated to single-end sequencing, the mapping results were

formatted in a bed6 format (bamToBed, BedTools [11]) on separate strands and then associated using a home-made bash script to place the R2 read on the same strand as its R1 companion. The DETR'PROK parameter values were set as follows: NC_009089.1.gff as annotation file and the deduced list of features concerning annotated loci (CDS, ncRNA, riboswitch, RNase_P_RNA, rRNA, SRP_RNA, tmRNA, tRNA), a read length of 100, op_gap 60, clust_gap 20, RNA_gap 25, RNA_merge 25, and, for Pruss/Fletcher studies and present study sRNA_coverage 3 and 2, asRNA_coverage 100 and 10, and 5utr_coverage 5 and 3, respectively, in order to adapt the coverage value to the different sequencing depths of the experiments.

References

1. Shelby RD, Tengberg N, Conces M, Olson JK, Navarro JB, Bailey MT, et al. Development of a Standardized Scoring System to Assess a Murine Model of *Clostridium difficile* Colitis. *Journal of Investigative Surgery* 2020; **33**: 887–895.
2. Bolger AM, Lohse M, Usadel B. Trimmomatic: a flexible trimmer for Illumina sequence data. *Bioinformatics* 2014; **30**: 2114–2120.
3. Kopylova E, Noé L, Touzet H. SortMeRNA: fast and accurate filtering of ribosomal RNAs in metatranscriptomic data. *Bioinformatics* 2012; **28**: 3211–3217.
4. Kim D, Paggi JM, Park C, Bennett C, Salzberg SL. Graph-based genome alignment and genotyping with HISAT2 and HISAT-genotype. *Nat Biotechnol* 2019; **37**: 907–915.
5. Langmead B, Salzberg SL. Fast gapped-read alignment with Bowtie 2. *Nat Methods* 2012; **9**: 357–359.
6. Danecek P, Bonfield JK, Liddle J, Marshall J, Ohan V, Pollard MO, et al. Twelve years of SAMtools and BCFtools. *Gigascience* 2021; **10**: giab008.
7. Liao Y, Smyth GK, Shi W. featureCounts: an efficient general purpose program for assigning sequence reads to genomic features. *Bioinformatics* 2014; **30**: 923–930.
8. Trivedi UH, Cézard T, Bridgett S, Montazam A, Nichols J, Blaxter M, et al. Quality control of next-generation sequencing data without a reference. *Front Genet* 2014; **5**.

9. Boudry P, Piattelli E, Drouineau E, Peltier J, Boutserin A, Lejars M, et al. Identification of RNAs bound by Hfq reveals widespread RNA partners and a sporulation regulator in the human pathogen *Clostridioides difficile*. *RNA Biology* 2021; 1–22.

10. Varet H, Brillet-Guéguen L, Coppée J-Y, Dillies M-A. SARTools: A DESeq2- and EdgeR-Based R Pipeline for Comprehensive Differential Analysis of RNA-Seq Data. *PLoS ONE* 2016; **11**: e0157022.

11. Quinlan AR, Hall IM. BEDTools: a flexible suite of utilities for comparing genomic features. *Bioinformatics* 2010; **26**: 841–842.
