# Supplementary material for: Dual RNA-seq study of the dynamics of coding and non-coding RNA expression during Clostridioides difficile infection in a mouse model
Source: mSystems. 2024 Nov 27;9(12):e00863-24. doi: 10.1128/msystems.00863-24 (PMC11651100; doi:10.1128/msystems.00863-24)
Supplement: Supplemental Tables — Tables S1, S3, S4, S7, and S8. [file msystems.00863-24-s0003.pdf]

Table S1 – Quantification of *C. difficile* vegetative cells in cecal content of infected mice.

Time of infection	UFC/g of cecal content	Mean
**8 h**	1.46E+08	**1.63E+08**
	2.31E+08	
	1.12E+08	
**28 h**	1.63E+08	**1.41E+08**
	1.42E+08	
	1.18E+08	
**32 h**	4.50E+06	**9.67E+07**
	2.46E+08	
	4.00E+07	

Table S2 – Differential Expression Analysis of *C. difficile* genes between late infectious conditions (28h and 32h post-infection) and *in vitro* cultures (excel format in a separate file).

Table S3 – Enrichment analysis of Ma2HTML classes with *C. difficile* expression profiles in infected mice *versus in vitro* growth conditions.

**Term (down)**	**nes**	**pval**	**fdr**	**leading_edge/geneset_size**
REGULATIONS	-6.44	1.19e-10	2.39e-09	160/338
STRESS	-4.59	4.42e-06	2.95e-05	38/97
MEMBRANE_TRANSPORT	-4.45	8.36e-06	4.18e-05	122/480
METABOLISM_NUCLEIC_ACID	-1.95	0.05	0.12	55/210
CELL_FACTOR	-1.10	0.26	0.45	72/244
CELL_GROWTH	-0.68	0.49	0.69	20/37
METABOLISM_COFACTOR	-0.65	0.51	0.69	44/101
FERMENTATION	-0.36	0.72	0.82	15/56
SECRETION	-0.22	0.82	0.87	15/34

**Term (up)**	**nes**	**pval**	**fdr**	**leading_edge/geneset_size**
SPORULATION	6.32	2.57e-10	2.57e-09	56/95
METABOLISM_LIPIDS	3.17	0.0015	0.0059	19/45
RESPIRATION_ANAEROBIC	2.3901	0.016	0.055	26/86

VIRULENCE_FACTORS	1.95	0.05	0.12	3/15
METABOLISM_AMINO_ACID	1.91	0.05	0.12	63/171
CELL_WALL	1.84	0.065	0.13	68/285
TRANSLATION	1.40	0.16	0.29	40/103
MOBILITY	0.69	0.49	0.68	21/53
MOBILE_ELEMENT	0.49	0.62	0.78	68/179
OPERONS_AUTOMATIC	0.34	0.73	0.82	1/51
METABOLISM_CARBON	0.0	1.0	1.0	15/147

Term : class name ; nes : normalized enrichment score ; pval : adjusted *p*-value ; fdr : false discovery rate ; leading_edge/geneset_size : number of leading genes / gene-set size

Table S4 – List of links for downloading of RNA-seq data from previously reported studies

Fletcher *et al*:

label	SRR_Id	condition	download link (*)
wt_d2_3	SRR11296256	wtd2	ftp.sra.ebi.ac.uk/vol1/fastq/SRR112/056/SRR11296256/
wt_d2_2	SRR11296257	wtd2	ftp.sra.ebi.ac.uk/vol1/fastq/SRR112/057/SRR11296257/
wt_d2_6	SRR11296259	wtd2	ftp.sra.ebi.ac.uk/vol1/fastq/SRR112/059/SRR11296259/
wt_d2_5	SRR11296270	wtd2	ftp.sra.ebi.ac.uk/vol1/fastq/SRR112/070/SRR11296270/
wt_d2_4	SRR11296281	wtd2	ftp.sra.ebi.ac.uk/vol1/fastq/SRR112/081/SRR11296281/
wt_TY_3	SRR11296276	wtTY	ftp.sra.ebi.ac.uk/vol1/fastq/SRR112/076/SRR11296276/
wt_TY_2	SRR11296277	wtTY	ftp.sra.ebi.ac.uk/vol1/fastq/SRR112/077/SRR11296277/
wt_TY_1	SRR11296278	wtTY	ftp.sra.ebi.ac.uk/vol1/fastq/SRR112/078/SRR11296278/

Pruss *et al*:

labels	SRR_Id	condition	download link (*)
WT4 WT_RNA_4	SRR12762560	WT	ftp.sra.ebi.ac.uk/vol1/fastq/SRR127/060/SRR12762560/
WT3 WT_RNA_3	SRR12762561	WT	ftp.sra.ebi.ac.uk/vol1/fastq/SRR127/061/SRR12762561/
WT2 WT_RNA_2	SRR12762562	WT	ftp.sra.ebi.ac.uk/vol1/fastq/SRR127/062/SRR12762562/

WT1 WT_RNA_1	SRR12762563	WT	ftp.sra.ebi.ac.uk/vol1/fastq/SRR127/063/SRR12762563/
Base3 IV3	SRR12766943	Base	ftp.sra.ebi.ac.uk/vol1/fastq/SRR127/043/SRR12766943/
Base2 IV2	SRR12766946	Base	ftp.sra.ebi.ac.uk/vol1/fastq/SRR127/046/SRR12766946/
Base1 IV1	SRR12766947	Base	ftp.sra.ebi.ac.uk/vol1/fastq/SRR127/047/SRR12766947/

(*) add the SSR_Id value followed by "_1.fastq.gz" and then "_2.fastq.gz" to get the complete links for downloading of the 2 files of the paired-end sequencing

Table S5 – Comparison with available *C. difficile in vivo* transcriptomic data (excel format in a separate file)

Table S6 - Prediction of new transcripts from RNA-seq data with DETR'PROK (excel format in separate file)

Table S7 - Functional analysis of host genes induced during CDI: genes overexpressed in infected mice (MI32H) compared to control mice (MC8H) (fc, fold change, > 2; p, p-value, <0.05; Cluster, cluster number as indicated in Figure 6, the fold changes are those between 32h-infected mice and control mice, but similar results were also obtained between 28h-infected mice and controls).

Gene ID	Description	Fold change	Cluster
**Host inflammatory markers**			
CCL4	chemokine (C-C motif) ligand 4	53.11	I
IL1b	interleukin 1 beta	51.15	I
CSCL1	chemokine (C-X-C motif) ligand 1	48.00	I
NLRP3	NLR family. pyrin domain containing 3	23.79	I
TNF	tumor necrosis factor	9.58	I
MEFV	Mediterranean fever	8.89	I
C5AR1	complement component 5a receptor 1	8.27	I
NFIL3	nuclear factor. interleukin 3. regulated	5.22	I
CEBPb	CCAAT/enhancer binding protein (C/EBP). beta	4.55	I
CCRL2	chemokine (C-C motif) receptor-like 2	4.33	I
NFKBIA	nuclear factor of kappa light polypeptide gene enhancer in B cells inhibitor. alpha	4.18	I
FOS	FBJ osteosarcoma oncogene	3.02	I
**Cell division and DNA repair**			
NUPR1	nuclear protein transcription regulator 1	6.86	III
PLK2	polo like kinase 2	3.55	III

POLR2K	polymerase (RNA) II (DNA directed) polypeptide K	2.59	III
CDC42EP 1	CDC42 effector protein (Rho GTPase binding) 1	2.54	III
UXT	ubiquitously expressed prefoldin like chaperone	2.27	III
NELFE	negative elongation factor complex member E. Rdbp	2.14	III
NCBP2	nuclear cap binding protein subunit 2	2.10	III
EXO1	exonuclease 1	2.04	III

Table S8 - Functional analysis of host genes repressed during CDI: genes repressed in infected mice (MI32H) compared to control mice (MC8H) (fc, fold change, < -2; p, p-value, <0.05; Cluster, cluster number as indicated in Figure 6)

**Gene ID**	**Description**	**Fold change**	**Cluster**
**Metabolism**			
ANPEP	alanyl (membrane) aminopeptidase	-8.94	VII
ABCC2	ATP-binding cassette. sub-family C (CFTR/MRP). member 2	-4.46	VII
NDST4	N-deacetylase/N-sulfotransferase (heparin glucosaminyl)	-4.05	VIII
GALNT13	polypeptide N-acetylgalactosaminyltransferase 13	-3.99	VIII
GALNTl6	UDP-N-acetyl-alpha-D-galactosamine:polypeptide N-acetylgalactosaminyltransferase-like 6	-3.62	VIII
GALNT14	polypeptide N-acetylgalactosaminyltransferase 14	-3.48	VIII
GAD2	glutamic acid decarboxylase 2	-3.26	VII
COL11A1	collagen. type XI. alpha 1	-3.17	VII
FBN2	fibrillin 2	-2.87	VII
HPN	hepsin	-2.65	VII
HPGD	hydroxyprostaglandin dehydrogenase 15 (NAD)	-2.49	VII
ANXA13	annexin A13	-2.48	VII
SELENOP	selenoprotein P	-2.45	VII
HAO2	hydroxyacid oxidase 2	-2.30	VII

DPP4	dipeptidylpeptidase 4	-2.29	VII
UGDH	UDP-glucose dehydrogenase	-2.19	VII
NQO1	NAD(P)H dehydrogenase. quinone 1	-2.01	VII
**Cell adhesion molecule**			
NRXN3	neurexin III	-5.19	VIII
CDH15	cadherin 15	-4.79	VIII
CLDN14	claudin 14	-4.58	VIII
CDH4	cadherin 4	-4.37	VIII
CNTNAP2	contactin associated protein-like 2	-3.67	VIII
NLGN1	neuroligin 1	-3.19	VIII
NRXN1	neurexin I	-2.97	VIII
CNTN1	contactin 1	-2.69	VIII
NCAM2	neural cell adhesion molecule 2	-2.65	VIII
NFASC	neurofascin	-2.54	VIII
JAM3	junction adhesion molecule 3	-2.42	VIII
MYH15	myosin. heavy chain 15	-3.52	VIII
CTNNA2	catenin (cadherin associated protein). alpha 2	-3.21	VIII
CTNNA 3	catenin (cadherin associated protein). alpha 3	-2.80	VIII
MYH8	myosin. heavy polypeptide 8. skeletal muscle. perinatal	-2.56	VIII
MAGI2	membrane associated guanylate kinase. WW and PDZ domain containing 2	-2.56	VIII
MYH7	myosin. heavy polypeptide 7. cardiac muscle. beta	-2.53	VIII
MYH 3	myosin. heavy polypeptide 3. skeletal muscle. embryonic	-2.35	VIII
MYH b	myosin. heavy chain 7B. cardiac muscle. beta	-2.21	VIII
**Signal transduction**			
NPFFR2	neuropeptide FF receptor 2	-9.13	VIII
GRIN1	glutamate receptor. ionotropic. NMDA1 (zeta 1)	-6.00	VIII
GRIN2b	glutamate receptor. ionotropic. NMDA2B (epsilon 2)	-5.26	VIII
ERBB4	erb-b2 receptor tyrosine kinase 4	-4.51	VIII

ATP2B2	ATPase. Ca++ transporting. plasma membrane 2	-4.5	VIII
GPR156	G protein-coupled receptor 156	-4.38	VIII
ADRA1b	adrenergic receptor. alpha 1b	-4.34	VIII
ATP2b3	ATPase. Ca++ transporting. plasma membrane 3	-4.17	VIII
GRIK4	glutamate receptor. ionotropic. kainate 4	-4.00	VIII
GALNT13	polypeptide N-acetylgalactosaminyltransferase 13	-3.98	VIII
ATP7b	ATPase. Cu++ transporting. beta polypeptide	-3.92	VIII
FSHR	follicle stimulating hormone receptor	-3.78	VIII
GRM8	glutamate receptor. metabotropic 8	-3.74	VIII
GRIA1	glutamate receptor. ionotropic. AMPA1 (alpha 1)	-3.74	VIII
HTR2C	5-hydroxytryptamine (serotonin) receptor 2C	-3.69	VIII
GRM3	glutamate receptor. metabotropic 3	-3.61	VIII
GALNTL6	UDP-N-acetyl-alpha-D-galactosamine:polypeptide N-acetylgalactosaminyltransferase-like 6	-3.61	VIII
GRM1	glutamate receptor. metabotropic 1	-3.54	VIII
GALNT14	polypeptide N-acetylgalactosaminyltransferase 14	-3.47	VIII
GRIK1	glutamate receptor. ionotropic. kainate 1	-3.43	VIII
GABRG2	gamma-aminobutyric acid (GABA) A receptor. subunit gamma 2	-3.43	VIII
RXFP1	relaxin/insulin-like family peptide receptor 1	-3.43	VIII
TSHR	thyroid stimulating hormone receptor	-3.38	VIII
GRM5	glutamate receptor. metabotropic 5	-3.35	VIII
PRLR	prolactin receptor	-3.30	VIII
GLDC	glycine decarboxylase	-3.28	VIII
CACNG2	calcium channel. voltage-dependent. gamma subunit 2	-3.22	VIII
CACNA1G	calcium channel. voltage-dependent. T type. alpha 1G subunit	-3.21	VIII
LHCGR	luteinizing hormone/choriogonadotropin receptor	-3.14	VIII
ATP8B3	ATPase. class I. type 8B. member 3	-3.11	VIII
GABRG3	gamma-aminobutyric acid (GABA) A receptor. subunit gamma 3	-3.08	VIII

GLIS1	GLIS family zinc finger 1	-3.08	VIII
GLRA2	glycine receptor. alpha 2 subunit	-3.03	VIII
CACNB4	calcium channel. voltage-dependent. beta 4 subunit	-2.94	VIII
